# Development of a novel chimeric lysin to combine parental phage lysin and cefquinome for preventing sow endometritis after artificial insemination

**DOI:** 10.1186/s13567-025-01457-4

**Published:** 2025-02-11

**Authors:** Xin-xin Li, Zi-qiang Hong, Zhi-xuan Xiong, Li-wen Zhang, Shuang Wang, Pan Tao, Pin Chen, Xiang-min Li, Ping Qian

**Affiliations:** 1 National Key Laboratory of Agricultural Microbiology, Hubei Hongshan Laboratory, Huazhong Agricultural University, Wuhan, 430070 Hubei China; 2 College of Veterinary Medicine, Huazhong Agricultural University, Wuhan, 430070 Hubei China; 3 Key Laboratory of Preventive Veterinary Medicine in Hubei Province, The Cooperative Innovation Center for Sustainable Pig Production, Wuhan, 430070 Hubei China

**Keywords:** Endometritis, chimeric lysin, phage lysin cocktail, antibiotic resistance, artificial insemination

## Abstract

Sow endometritis is usually caused by multiple species of pathogenic bacteria. Numerous isolates from endometritis patients have developed antimicrobial resistance. Thus, novel antibacterial agents and strategies to combat endometritis are needed. A total of 526 bacteria, including *Staphylococcus* spp. (26.3%), *Streptococcus* spp. (12.3%), *E. coli* (28.9%), *Enterococcus* spp. (20.1%), *Proteus* spp. (9.5%), and *Corynebacterium* spp. (2.8%), were isolated from sows with endometritis. We constructed a novel chimeric lysin, ClyL, which is composed of a cysteine- and histidine-dependent amidohydrolase/peptidase (CHAP) catalytic domain from the phage lysin LysGH15 and a cell wall-binding domain (CBD) from the prophage lysin Lys0859. The activities of ClyL and Lys0859 were most pronounced for the *Staphylococcus* and *Streptococcus* strains isolated from sow endometritis and bovine mastitis, respectively. ClyL and Lys0859 were combined to create a phage lysin cocktail, which demonstrated a synergistic effect against the coinfection of *Staphylococcus* and *Streptococcus* in vitro and in vivo. Furthermore, the combination of phage lysin cocktail and cefquinome had a synergistic bactericidal effect on boar semen that did not influence the activity of sperm. Remarkably, the incidence rate of sow endometritis was 0% (0/7) when the combination of phage lysin cocktail and cefquinome was used in semen via artificial insemination compared with 50% (3/6) when PBS was administered. Overall, the administration of a phage lysin cocktail and cefquinome in semen via artificial insemination is a promising novel strategy to prevent sow endometritis after artificial insemination.

**Supplementary Information:**

The online version contains supplementary material available at 10.1186/s13567-025-01457-4.

## Introduction

Endometritis, an inflammatory disease, seriously affects the reproductive performance of sows (including the sensitivity of piglets to infection, growth, and mortality) and leads to enormous economic losses [1–3]. It is caused predominantly by common bacteria (including *Staphylococcus aureus*, *Streptococcus* spp., and *Escherichia coli*), viruses (including Japanese encephalitis, porcine reproductive, and respiratory syndrome virus), and parasites (e.g., Trichomonas) [3–6]. Ye et al. isolated a total of 556 bacterial strains from 200 sows across 11 farms located in Guangdong and Jiangsu Provinces, China. *S. aureus* was the most prevalent isolate (34%, *n* = 188), followed by *Streptococcus* (32%, *n* = 181), *E. coli* (19%, *n* = 104) and other bacilli (15%, *n* = 83) [3]. A significant proportion of sow endometritis cases (83%, *n* = 207) were identified as mixed infections, complicating clinical treatment efforts [3]. The microbiota results of birth canal secretions and fresh feces from healthy sows and sows with endometritis revealed that the abundances of *Escherichia–Shigella* and *Streptococcus* in sows suffering from endometritis were significantly greater than those in healthy sows [1]. Bacterial-induced endometritis has a significant effect on the reproductive capabilities of both animals and humans, contributing to the death of 6.6% of breeding sows on large-scale farms [7–9]. Cow endometritis can lead to embryo transfer failure [8]. Antibiotic therapy can significantly improve the rate of successful pregnancies in women with CE undergoing IVF [10]. Similarly, the pregnancy rate of the mare was significantly improved by intrauterine infusion of broad-spectrum antibiotics compared with that of the untreated group (*P* < 0.01) [11]. However, isolates from animals and humans with endometritis commonly exhibit antimicrobial resistance (AMR) [2, 12, 13], and AMR has become a global health concern that has been further exacerbated by the slow development of novel antibiotics. Previous research has shown that lysostaphin can be used to treat clinical sow endometritis via intravaginal administration [3]. Following treatment with lysostaphin at a dosage of 400 U, the average cure rate of lysostaphin on sow endometritis (82.5%) was greater than that of the antibiotic group (72.17%) [3]. Phage lysin and chimeric lysin, as novel antibiotic alternatives, can rapidly lyse bacteria and not induce bacterial resistance to phage lysin [14–16]. At present, many bacterial diseases, including endometritis, osteomyelitis, and bovine mastitis, are induced by various bacterial species. However, to our knowledge, it is difficult for a phage lysin to efficiently lyse multiple genera of bacteria. For example, the *S. aureus* phage lysin LysGH15 has potent activity specifically targeting *Staphylococcus* spp. but has no discernible antibacterial efficacy against other bacterial strains [17–19]. Similarly, the chimeric lysin Csl2 possesses antibacterial activity against *Streptococcus* but not *S. aureus* [20]. To address these challenges, we envision that novel chimeric lysins can be exploited and applied to address the lack of host range of parental phage lysins. The chimeric lysin and parental phage lysin can be composed of a phage lysin cocktail against multiple bacterial infections. Furthermore, the broad-spectrum activity of antibiotics can be a suitable component. It has been widely proposed that the combination of phage lysin and antibiotics is superior to phage lysin and antibiotic monotherapy for treating bacterial infections [21–23] and can be applied for the treatment of bacterial infections in humans and animals [24, 25]. Additionally, our previous study revealed that the chimeric lysin ClyQ does not induce resistance in *S. aureus* and that the combination of ClyQ and mupirocin can delay the development of mupirocin resistance [26]. In conclusion, the combination of a phage lysin cocktail and antibiotics may be a solution for multibacterium-induced infections. However, the efficacy of a combination of antibiotics and phage lysin cocktails for the prevention of bacterium-induced endometritis remains to be elucidated.

In this study, a total of 526 bacteria, including *Staphylococcus* spp. (26.3%), *Streptococcus* spp. (12.3%), *E. coli* (28.9%), *Enterococcus* spp. (20.1%), *Proteus* spp. (9.5%), and *Corynebacterium* spp. (2.8%), were isolated from 383 vaginal swab samples from sows with endometritis, and the antibiotic resistance of *Staphylococcus* and *Streptococcus* was tested. Moreover, we constructed and purified a novel broad-host-range chimeric lysin, ClyL, which possessed excellent antibacterial activity against *Staphylococcus* app. Moreover, the prophage lysin Lys0859 was reported in our previous study and was shown to have exceptional activity against *Streptococcus* app [27]. ClyL and Lys0859 are composed of a phage lysin cocktail and exhibit synergistic effects against dual-species biofilms, *Galleria mellonella* larvae, and mouse mastitis models caused by coinfection with *Staphylococcus* and *Streptococcus*. Finally, the phage lysin cocktail was combined with cefquinome to form a combination therapy. We evaluated the effects of combination therapy on the activity of semen and the effects of semen on the bactericidal activity of combination therapy. Additionally, to prevent sow endometritis, we utilized semen as a vector to transport the combination of phage lysin cocktail and cefquinome by artificial insemination (AI).

## Materials and methods

### Collection of sow endometritis samples and microbiological analysis

Sow endometritis samples were collected as previously described [4, 28]. A total of 383 vaginal swab samples were collected from sows suffering from endometritis on 24 large-scale sow farms between April and August 2022 in Guigang city, Guangxi Province, Southern China. The sows that developed endometritis within five days following their first to sixth deliveries were selected for this study. These sows presented symptoms of fever and anorexia, and their vulvas were erythematous and oedematous. Additionally, purulent or mucopurulent uterine exudates are present in the vaginal canal of affected sows [1, 2]. To avoid contamination of the vaginal samples during sampling, special swab equipment was used to collect the samples. The structure of the swab equipment was the same as previously described [4]. All the samples were immediately placed on dry ice and transported to the laboratory. Afterwards, the samples were plated on tryptic soy agar (TSA) plates, which were incubated at 37 °C for 24–48 h. Single colonies of various shapes and sizes were selected and purified.

The strains were inoculated into tryptic soy broth (TSB) and cultured for 18–24 h at 37 °C, after which the bacterial DNA was extracted using a Bacterial DNA Extraction Kit (Tiangen Biotech, China) following the manufacturer’s instructions (Tiangen Biotech, China). The concentrations of extracted DNA were determined using a spectrophotometer (Thermo Scientific, USA). All the DNA samples were stored at −20 °C until further use. The bacteria were identified by the previously described primer sequences (Additional file 1) and sequenced by Guangzhou Ige Biotechnology Ltd. (Guangzhou, China), and the sequences were compared with the 16S rDNA sequence in the NCBI (National Center for Biotechnology Information) public database using the BLAST program (Basic Local Alignment Search Tool). The serotypes of *Streptococcus suis* were determined as previously described [29]. The related primers are listed in Additional file 2.

### Antimicrobial susceptibility testing

The minimal inhibitory concentrations (MICs) of the isolated *E. coli*, *Staphylococcus*, and *Streptococcus* strains were determined according to the Clinical Laboratory and Standards Institute (CLSI) 2018 [30], and the breakpoints of antibiotics against *E. coli*, *Staphylococcus*, and *Streptococcus* are presented in Additional file 3. A total of fifteen antibiotics were selected, including ampicillin, amoxicillin, penicillin G, ceftiofur, cefquinome, gentamicin, tetracycline, doxycycline, florfenicol, chloramphenicol, amikacin, erythromycin, tilmicosin, lincomycin, and enrofloxacin. *S. aureus* ATCC 29213 and *Streptococcus pneumoniae* ATCC 49619 served as quality control strains. Finally, the MIC_50_ and MIC_90_ values were calculated as the MIC that inhibited 50% and 90% of the isolates, respectively.

### Expression and purification of ClyL and Lys0859

The protocols for constructing and purifying Lys0859 were reported in our previous study [27]. The gene encoding the full-length *S. aureus* phage lysin LysGH15 (GenBank: ADG26756.1) was synthesized by Tsingke Biotechnology Co., Ltd. ClyL was constructed from the putative catalytic domain CHAP (from LysGH15, amino acids 1–165) with a cell wall-binding domain (from Lys0859, amino acids 143–239). The primers used for the construction of ClyL are listed in Additional file 4. The expression and purification of ClyL were carried out as previously described [26]. Briefly, the pET-28a (+) _ClyL vector was transformed into *E. coli* BL21 (DE3) cells, which were subsequently grown to OD_600nm_ ~ 0.6 in LB containing 50 μg/mL kanamycin (37 °C, 200 g). The BL21 cells were subsequently cultured at 16 °C with 0.8 mM isopropyl-beta-d-thiogalactopyranoside (IPTG) for 16–18 h and then harvested by centrifugation at 6000 × *g* for 15 min. The cells were subsequently resuspended in binding buffer (250 mM NaCl, 20 mM Tris–HCl, pH 7.4), and the supernatants were collected after sonication at 4 °C. Lys0859 was purified by binding the 6× His-tagged fusion protein supernatant to a HisTrap FF column (GE Healthcare Bio-Sciences AB, USA) and eluted with elution buffer (250 mM NaCl, 20 mM Tris–HCl, 200 mM imidazole, pH 7.4) following the manufacturer’s instructions (AKTA pure 25 M, GE Healthcare Bio-Sciences AB, USA).

### Lytic activity of ClyL and Lys0859 toward *Streptococcus* and *Staphylococcus*

The lytic activity of ClyL and Lys0859 against *Streptococcus* and *Staphylococcus* was examined through turbidity reduction and bactericidal assays as previously described [27]. First, 100 μL of log-phase bacteria in PBS (OD_600nm_ = 0.6–0.8) was added to 100 μL of ClyL or Lys0859 in 96-well plates (NEST Biotechnology Co., Ltd., China), with PBS used as a negative control. The final concentrations of ClyL and Lys0859 were 50 μg/mL. After incubation at 37 °C for 30 min, the OD_600nm_ values of the mixtures, including bacteria and lysin, were measured. On the other hand, log-phase bacteria were added to ClyL and Lys0859 at 37 °C for 1 h, and the final concentrations of ClyL and Lys0859 were 50 μg/mL. Thereafter, the bacterial count in the suspension treated with lysin was calculated via plate counting of tenfold serial dilutions. All the experiments were performed in triplicate.

### Checkerboard assay

The checkerboard assay for the combination of ClyL and Lys0859 was performed in 96-well plates as previously described [31]. The concentration gradients of ClyL and Lys0859 were prepared in the horizontal and vertical directions. The log-phase *S. aureus* strains ATCC29213, *S. agalactiae* ATCC13813, and *Staphylococcus* 65 were diluted to 10^5^ cfu/mL, and the bacteria were added to each well. The plates were subsequently incubated at 37 °C for 16–18 h. Finally, the MIC was recorded. Moreover, the MIC of ClyL and Lys0859 alone against the above bacteria were also tested. The values of the fractional inhibitory concentration index (FICI) were calculated according to previous methods [32].

### Antibiofilm activity of ClyL and Lys0859

The antibiofilm ability of ClyL and Lys0859 was evaluated as previously described, with some changes [27]. *Staphylococcus* and *Streptococcus* strains were diluted to 1.0 × 10^5^ cfu/mL. Two hundred microliters of bacteria were subsequently added to 96-well plates, which were subsequently incubated for 24 and 48 h at 37 °C. Two hundred microlitres of PBS, ClyL (100 μg/mL), Lys0859 (100 μg/mL), ClyL (100 μg/mL) + Lys0859 (100 μg/mL), or ClyL (50 μg/mL) + Lys0859 (50 μg/mL) was then added to the 96-well plates and incubated at 37 °C for 2 h. The biofilm biomass was evaluated using crystal violet [1.0% (weight/vol)] staining, and the viable bacteria were counted on the TSB agar plates. In addition, to determine the number of viable bacteria in the dual-species biofilms, *S. aureus* ATCC29213 and *S. agalactiae* ATCC13813 were cultured on Baird‒Parker agar (BP, HuanKai Microbial, Guangzhou, China) and Edwards Medium agar (modified, Oxoid, UK), respectively [33]. Scanning electron microscopy (SEM) images of the dual-species biofilms were obtained as previously described [26]. *Staphylococcus* and *Streptococcus* strains were diluted to 1.0 × 10^5^ cfu/mL. *S. aureus* ATCC29213 and *S. agalactiae* ATCC13813 were mixed equivalently, and then, the mixtures were grown in media (TSB: PBS = 1:1) on 24-well plates with coverslips at 37 °C for 48 h. Thereafter, the 24-well plates were treated with PBS, ClyL (100 μg/mL), Lys0859 (100 μg/mL), and phage lysin cocktail ClyL (100 μg/mL) + Lys0859 (100 μg/mL) at 37 °C for 2 h. Finally, the images of the dual-species biofilms were observed by FESEM (2000× and 5000×) (NTC, Japan).

### The effectiveness of phage lysin cocktail on *G. mellonella* larvae and a mouse mastitis model of coinfection with *S. aureus* ATCC29213 and *S. agalactiae* ATCC13813

The *G. mellonella* larvae were injected with bacteria and phage lysin as previously described [34]. Larvae of *G. mellonella* were obtained from Guiling Jiacheng Co., Ltd. (Guangxi, China). To identify the minimum lethal dose (MLD100), *G. mellonella* larvae were infected with different doses of *S. aureus* ATCC29213 or *S. agalactiae* ATCC13813 (5 × 10^6^, 1 × 10^6^, 5 × 10^5^, or 1 × 10^5^ cfu/bacteria/larvae). The larvae were inoculated with *S. aureus* ATCC29213 and *S. agalactiae* ATCC13813 at a concentration of 5 × 10^6^ cfu/bacteria/larvae, followed by treatment with a combination of ClyL (1.5 μg/larvae) and Lys0859 (1.5 μg/larvae), ClyL (1.5 μg/larvae), Lys0859 (1.5 μg/larvae), or PBS. The survival rates of the larvae were observed at 72 h post infection. A mouse model of bovine mastitis was generated as previously described with some modifications [35]. Briefly, the L4 and R4 mammary glands were injected with 1 × 10^5^ cfu/g of *S. aureus* ATCC29213 or *S. agalactiae* ATCC13813. The mice were subsequently divided into four groups (*n* = 3), and the mice were injected with a combination of 20 μg/g of Lys0859 or ClyL, 20 μg/g of Lys0859, 20 μg/g of ClyL, or 50 μL of PBS at 24 h postinfection. The L4 and R4 mammary gland tissues were ground, serially diluted, and spread on TSA plates at 48 h postinfection.

### Assessment of the effects of the combination of the lysin cocktail and cefquinome on bacterial activity in semen

The lytic activity of ClyL, Lys0859, and cefquinome against bacteria was assessed in semen and TSB as previously described [36]. Briefly, *Staphylococcus* 65 was grown to the exponential growth phase. The bacterial suspension was subsequently centrifuged and washed twice with PBS. Finally, the bacteria were resuspended in TSB and commercial boar semen (Guangxi Yangxiang Co., Ltd.) and diluted to 10^6^ cfu/mL. Commercial boar semen includes boar semen and Zenolong® extender (Beikang, Taizhou, China). As commercial extenders, the specific composition of Zenolong® is not known. The concentration of sperm in commercial boar semen is 2.5 × 10^7^ spermatozoa/mL [37]. With respect to the phage lysin cocktail and cefquinome used in isolation, ClyL, Lys0859, and cefquinome were added to TSB. Next, the concentrations of ClyL and Lys0859 were adjusted to 50, 25, 12.5, and 0 μg/mL, and those of cefquinome were adjusted to 4, 2, 1 (2 μg/mL), and 0 × MIC. For the combination of the phage lysin cocktail and cefquinome, the phage lysin cocktail ClyL (25 μg/mL) + Lys0859 (12.5 μg/mL), cefquinome monotherapy (20 × MIC, 80 μg/mL), and ClyL (25 μg/mL) + Lys0859 (12.5 μg/mL) + cefquinome (20 × MIC) were added to TSB and semen. TSB and semen containing PBS and *Staphylococcus* 65 served as the control group. The mixtures were subsequently incubated at 37 °C for 24 h. Finally, the total number of bacterial colonies was counted at 0, 2, 4, 6, 8, 12, 16, 20, and 24 h. All the experiments were repeated three times.

### Interactions among phage lysin, cefquinome, and boar semen

The phage lysin cocktail ClyL (25 μg/mL) + Lys0859 (12.5 μg/mL) alone, cefquinome (20 × MIC, 80 μg/mL) alone, and ClyL (25 μg/mL) + Lys0859 (12.5 μg/mL) + cefquinome (20 × MIC, 80 μg/mL) were added to commercial boar semen. Next, the semen was stored at 17 °C. The antibacterial activity of the phage lysin cocktail alone, cefquinome alone, and ClyL + Lys0859 + cefquinome groups was examined at 4, 7, and 11 days. Specifically, the *Staphylococcus* 65 strain (10^6^ cfu/mL) was added to semen containing phage lysin cocktail, cefquinome, and ClyL + Lys0859 + cefquinome and incubated at 37 °C for 2 h. The viable bacteria were counted on TSB agar plates. Moreover, the total motility, progressive motility, velocity curved line (VCL), average path velocity (VAP), straight-line velocity (VSL), linearity (LIN), amplitude of lateral head displacement (ALH), wobble (WOB), and beat-cross frequency (BCF) of the sperm were observed by computer-aided sperm analysis (CASA) (IMV Technologies, France) as previously described [38, 39]. The acrosome integrity and MMP of the sperm were determined by the Viability and Acrosome Integrity Easykit 5 (IMV Technologies, France) and Mitochondrial Activity EasyKit 2 (IMV Technologies, France), respectively, as previously described [40].

### Efficacy of cefquinome combined with phage lysin cocktail in sow endometritis

Approximately 20 days postpartum, the sows were acquired from a commercial swine farm in Guangxi Province, China, and subsequently allocated into four experimental groups (*n* = 7). The veterinarian verified that these sows did not suffer from endometritis and only exhibited normal inflammation after delivery. The veterinarian also confirmed that the sows met the criteria for AI. The four groups of sows were treated with commercial semen twice within 24 h, which was mixed with PBS, ClyL (25 μg/mL) + Lys0859 (12.5 μg/mL), cefquinome (80 μg/mL) alone, or ClyL (25 μg/mL) + Lys0859 (12.5 μg/mL) + cefquinome (80 μg/mL) via AI. The clinical pregnancy rate, live birth rate, and recurrence endometritis rate were recorded at 4 months after AI.

### Statistical analysis

Statistical analyses were performed using GraphPad Prism (version 9). Statistical significance was determined using an unpaired Student’s *t* test. *P* < 0.05 was considered statistically significant. All the data are expressed as the means ± standard deviations (SD) of three independent experiments.

## Results

### Identification of sow endometritis isolates

A total of 526 bacterial strains were isolated from 383 vaginal swab samples. The 526 strains included *Staphylococcus* spp. (26.3%, 141/536), *Streptococcus* spp. (12.3%, 66/536), *E. coli* (28.9%, 155/536), *Enterococcus* spp. (20.1%, 108/536), *Proteus* spp. (9.5%, 51/536), and *Corynebacterium* spp. (2.8%, 15/536) (Table 1). Moreover, all the *Streptococcus* strains were identified as *S. suis* (Table 1). The proportions of all strains are listed in Table 1. Similarly, a previous study reported that the bacteria most frequently isolated from mares with endometritis were *E. coli* (17.3%), *Staphylococcus* spp. (15.6%), and *Streptococcus* spp. (13.5%) [41]. In addition, 556 strains, including *S. aureus* (34%, *n* = 188), *Streptococcus* (32%, *n* = 181), and *E. coli* (19%, *n* = 104), were collected from 200 sows infected with endometritis [3]. Taken together, the results of this study inferred that *E. coli*, *Staphylococcus*, and *Streptococcus* (*S. suis*) play a pivotal role in endometritis.

**Table 1:** **Prevalence of bacteria isolated from sow endometritis**

No	Genus	Species	Number of isolates	Percentage
1	*Staphylococcus* spp			(26.3%, 141/536)
2	*Streptococcus* spp	*S. suis*	66	(12.3%, 66/536)
3	*Escherichia*	*E. coli*	155	(28.9%, 155/536)
4	*Enterococcus* spp		108	(20.1%, 108/536)
5	*Proteus* spp		51	(9.5%, 51/536)
6	*Corynebacterium* spp		15	(2.8%, 15/536)

### Antibiotic susceptibility of sow endometritis isolates

Our initial step is to identify an antibiotic that is sensitive to bacteria and can effectively synergize with phage lysin to combat endometritis induced by multiple bacterial strains. The results of bacterial sensitivity to antimicrobial drugs can guide the utilization of antibiotics [41]. Therefore, the MICs of the fifteen antibiotics were tested for *Staphylococcus*, *S. suis*, and *E. coli*. On the basis of the existing criteria for antibiotics (Additional file 3), 63.1% (89/141), 85.5% (121/141), and 88.7% (125/141) of the *Staphylococcus* strains were insensitive to chloramphenicol, erythromycin, and lincomycin, respectively (Additional file 5). The findings regarding antibiotic resistance revealed that a significant proportion of *S. suis* strains, specifically 98.5% (65/66), 93.9% (62/66), and 100% (66/66), exhibited resistance to tetracycline, erythromycin, and lincomycin, respectively (Additional file 6). A total of 81.3% (126/155), 71.6% (111/155), 62.6% (97/155), 69.7% (108/155), and 71.0% (110/155) of the *E. coli* were resistant to ampicillin, tetracycline, doxycycline, florfenicol and chloramphenicol, respectively (Additional file 7).

### Construction and expression of the chimeric lysin ClyL and effects of ions on the enzymatic activity of ClyL

**Figure 1:**
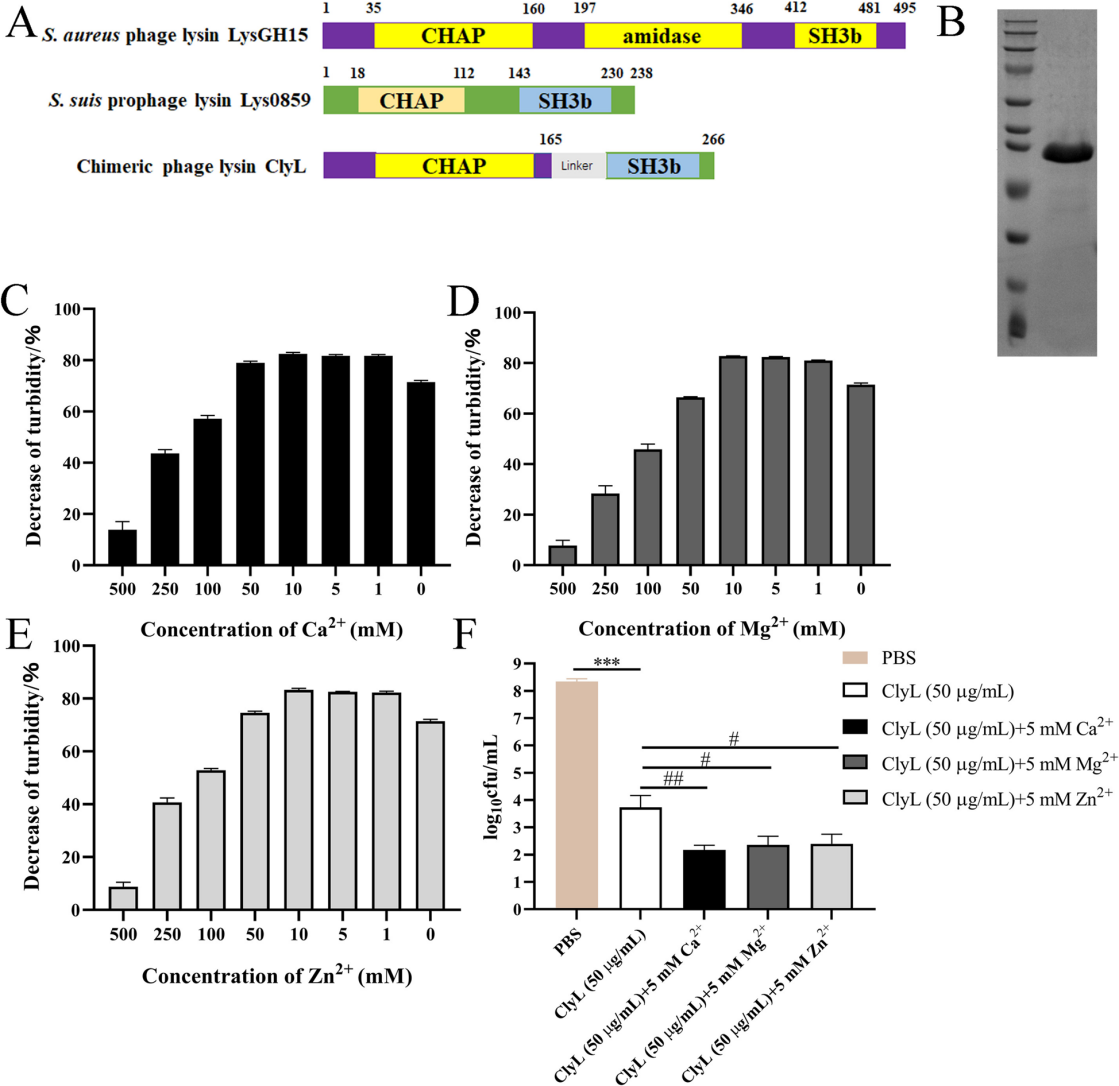
**Schematic representation and SDS analysis of ClyL.** **A** The novel chimeric lysin ClyL, which is composed of a cysteine- and histidine-dependent amidohydrolase/peptidase (CHAP) catalytic domain (1–165 aa) from the phage lysin LysGH15 and a cell wall-binding domain (CBD) (143–239 aa) from the prophage lysin Lys0859. **B** SDS‒PAGE of the recombinant chimeric lysin ClyL. M: marker; 1: purified ClyL (34.0 kDa). **C**–**F** Effects of metal ions on the antibacterial activity of Lys0859. Different concentrations of Ca^2+^ (**C**), Mg^2+^ (**D**), and Zn^2+^ (**E**) were added to ClyL (50 μg/mL), and then the mixtures were mixed with *S. aureus* ATCC 29213 at 37 °C for 30 min. **E** PBS and 5 mM Ca^2+^, Mg^2+^, and Zn^2+^ were added to Lys0859 (50 μg/mL), and the mixtures were mixed with *S. aureus* ATCC 29213 at 37 °C for 60 min. Significant differences between the PBS groups and the ClyL groups were determined by Student’s *t* test (*** *P* < 0.001). Significant differences between the ClyL groups and the ClyL + 5 mM Ca^2+^, ClyL + 5 mM Mg^2+^, and ClyL + 5 mM Zn^2+^ groups were determined by Student’s *t* test (# *P* < 0.05, ## *P* < 0.05). The data are expressed as the mean ± standard deviation (SD).

On the basis of the results of antibiotic susceptibility, *E. coli, Staphylococcus,* and *Streptococcus* (*S. suis*) isolated from sow endometritis samples were sensitive to cefquinome. Therefore, it is imperative to exploit phage lysin, which can efficiently lyse both *Staphylococcus* and *S. suis,* for the successful implementation of the proposed combined therapy. Our previous study demonstrated that Lys0859 displayed excellent activity against 15 serotypes of *S. suis* [27]. Moreover, the *S. aureus* phage lysin LysGH15 has exceptional effects on *Staphylococcus* [17–19, 42]. Hence, to obtain an antistaphylococcal phage lysin, we constructed a new chimeric lysin, ClyL, which is composed of the catalytic domain CHAP from LysGH15 and a CBD of the prophage lysin Lys0859 (Figure 1A). The molecular weight of ClyL was approximately 34.0 kDa (Figure 1B). The stability of the novel chimeric lysin ClyL was tested in the presence of various metal ions. The results of the turbidity reduction assay indicated that varying concentrations of Ca^2+^ (1–50 mM), Mg^2+^ (1–10 mM), and Zn^2+^ (1–50 mM) have the potential to enhance the lytic activity of ClyL, as depicted in Figure 1C-E. Additionally, a heightened bactericidal effect of ClyL was noted in PBS buffer with 5 mM concentrations of Ca^2+^, Mg^2+^, and Zn^2+^ (Figure 1F). Similarly, Ca^2+^ promotes the activity of the chimeric lysin ClyC in a dose-dependent manner (1–1000 μM) [43]. Research has shown that the lytic activity of the LysGH15CHAP domain is specifically dependent on calcium [18]. The findings of our study indicated that Mg^2+^, Zn^2+^, and Ca^2+^ were capable of enhancing the bactericidal efficacy of ClyL (Figure 1C–F). We previously reported that Lys0859 exhibited increased activity against *S. suis* in PBS buffer containing Mg^2+^ and Ca^2+^ but had little lytic activity in PBS buffer containing Zn^2+^ [27].

### *Staphylococcus* and* Streptococcus* strains isolated from sow endometritis and bovine mastitis samples are sensitive to ClyL and Lys0859 in vitro

**Figure 2:**
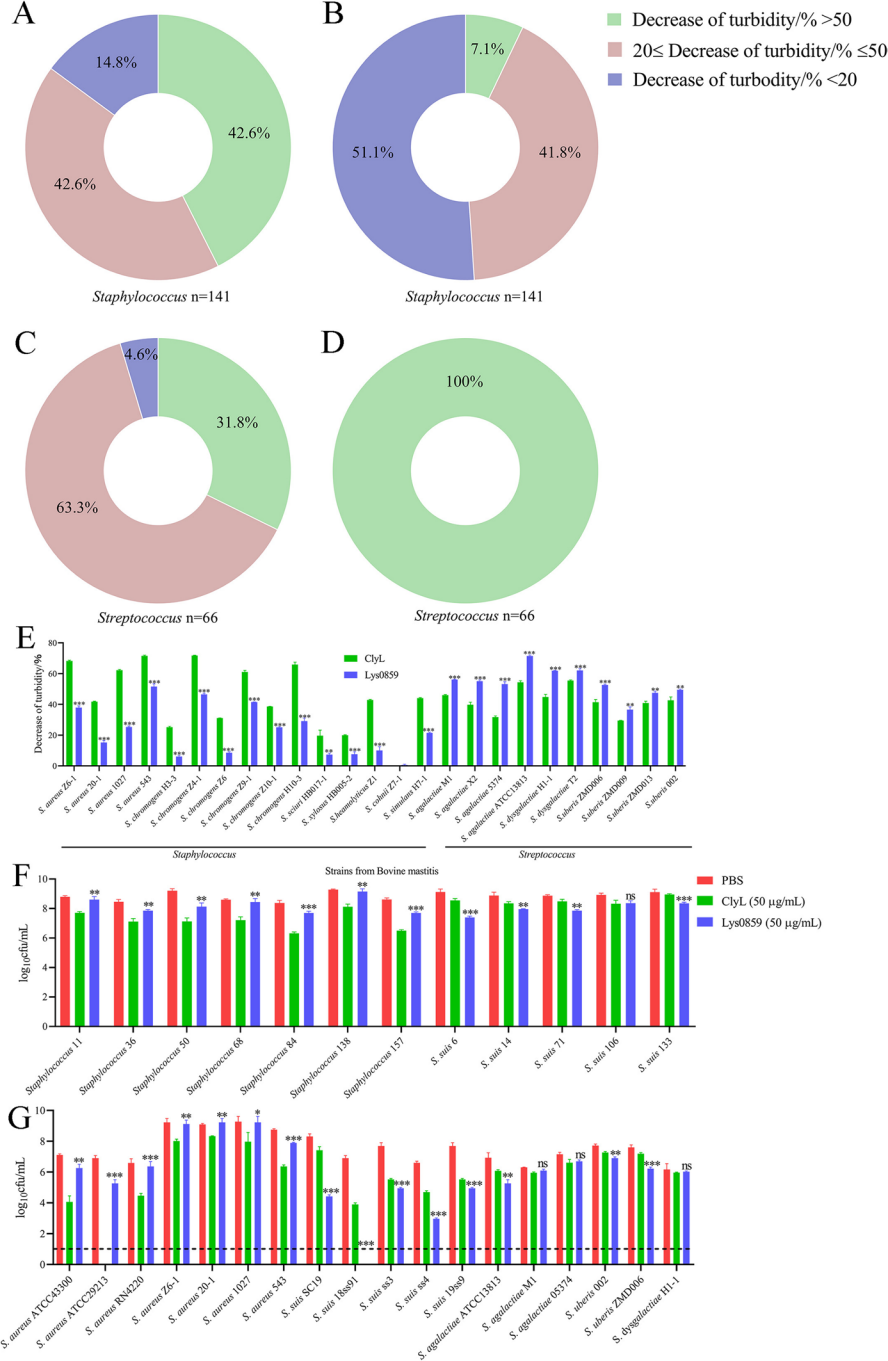
**The phages lysin ClyL and Lys0859 could effectively lyse bacteria isolated from sows with endometritis and bovine mastitis.** **A**–**D** The antibacterial activity of Lys0859 (50 μg/mL) and ClyL (50 μg/mL) against clinical bacteria isolated from sows subjected to endometritis at 37 °C for 1 h. The values of turbidity decrease were classified into three parts: a decrease in turbidity > 50%, a 20% ≤ decrease in turbidity ≤ 50%, and a decrease in turbidity < 20%. The proportions of the three parts are presented in a circular figure. The proportions of the decrease in ClyL turbidity against *Staphylococcus* and *Streptococcus* are listed in A and C, respectively. The proportions of Lys0859 turbidity decreases against *Staphylococcus* and *Streptococcus* are listed in **B** and **D**, respectively. **E** The antibacterial activity of Lys0859 (50 μg/mL) and ClyL (50 μg/mL) against clinical bacteria isolated from bovine mastitis. **F**, **G** The bacterial count test was used to evaluate the bactericidal activity of Lys0859 (50 μg/mL) and ClyL (50 μg/mL) against *Staphylococcus* and *Streptococcus* strains isolated from sows with endometritis (**F**) and bovine mastitis (**G**). Significant differences between the Lys0859 groups and ClyL groups were determined by Student’s *t* test (** *P* < 0.01, and *** *P* < 0.001). The data represent the mean ± SD of three independent experiments.

First, *S. suis* and *Staphylococcus* strains isolated from sow endometritis were utilized to assess the lytic activity of ClyL and Lys0859. As depicted in Figure 2A and C, the turbidity of *Staphylococcus* (85.2%) and *S. suis* (95.4%) could be decreased by 20% or more by ClyL. Similarly, the turbidity of *Staphylococcus* (48.9%) and *S. suis* (100%) could be decreased by greater than 20% by Lys0859 (Figure 2B and D). Details of turbidity are shown in Additional files 8 and 9. In addition, bovine mastitis is induced by multiple bacterial species, and staphylococcal and streptococcal strains are the main bacterial pathogens [44]. Thus, we utilized staphylococcal and streptococcal strains from bovine mastitis to determine the bactericidal activity of ClyL and Lys0859. As shown in Figure 2E, ClyL showed exceptional antistaphylococcal activity compared with Lys0859. Moreover, compared with ClyL, Lys0859 exhibited significant antibacterial activity against *Streptococcus* (Figure 2E). Similarly, according to the bacterial count test, the activities of ClyL and Lys0859 were most pronounced for the *Staphylococcus* and *Streptococcus* strains isolated from sow endometritis and bovine mastitis, respectively (Figure 2F and G). To determine the synergistic effect of ClyL and Lys0859, we tested the FICI values of a combination of ClyL and the parental phage lysin Lys0859. The fractional inhibitory concentration index (FICI) values for *S. aureus* ATCC29213, *S. agalactiae* ATCC13813, and *Staphylococcus* 65 were determined to be 0.5, 0.266, and 0.266, respectively (Additional file 10). For the coinfection of *S. aureus* ATCC29213 and *S. agalactiae* ATCC13813, the FICI value was 0.281 (Additional file 10). On the basis of the established criteria for synergistic effects outlined previously [26, 32], ClyL and Lys0859 exhibit synergistic activity against strains of *Staphylococcus* and *Streptococcus*, as well as coinfections involving these bacterial strains.

### The combination of ClyL and Lys0859 has a synergistic effect on coinfection with *Staphylococcus* and *Streptococcus* in vitro and in vivo

**Figure 3:**
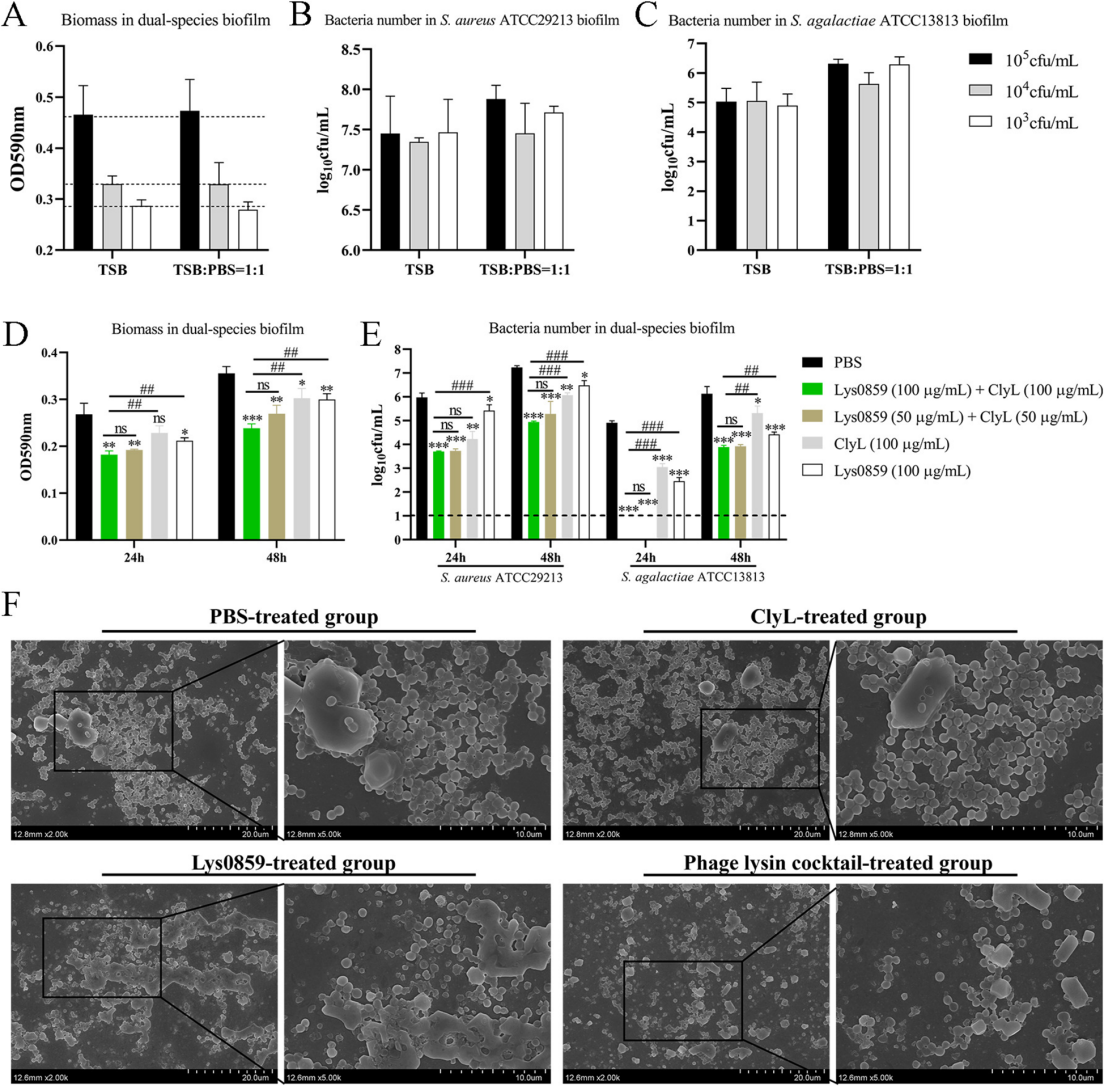
**Chimeric lysin ClyL and parental phage lysin Lys0859 were used to treat 24-h- and 48-h-old biofilms formed by *S. aureus* ATCC29213 and *S. agalactiae* ATCC13813 coinfection.** **A**–**C** The optimal conditions for dual-species biofilm formation. **A** The biomass of dual-species (*S. aureus* ATCC29213 and *S. agalactiae* ATCC13813) biofilms formed at 37 °C for 24 h. The viable cell counts of dual-species biofilms of *S. aureus* ATCC29213 (**B**) and *S. agalactiae* ATCC13813 (**C**) formed at 37 °C for 24 h. **D** The biomass of dual-species biofilms treated with ClyL, Lys0859, or ClyL + Lys0859 at 37 °C for 2 h. **E** Viable cell counts of dual-species biofilms treated with ClyL, Lys0859, and ClyL + Lys0859 at 37 °C for 2 h. Significant differences between the PBS groups and the ClyL, Lys0859, and ClyL + Lys0859 groups were determined via Student’s *t* test (ns *P* > 0.05, * *P* < 0.05 ** *p* < 0.01, and *** *P* < 0.001). Significant differences between the ClyL + Lys0859 groups and the ClyL and Lys0859 groups were determined via Student’s *t* test (ns *P* > 0.05, # *P* < 0.05, ## *P* < 0.01, and ### *P* < 0.001). The error bars represent the standard deviations (SD). **F** FESEM images of dual-species biofilms (48 h) formed by *S. aureus* ATCC29213 and *S. agalactiae* ATCC13813. The dual-species biofilms were treated with PBS, ClyL (100 μg/mL), Lys0859 (100 μg/mL), or the phage lysin cocktail ClyL (100 μg/mL) + Lys0859 (100 μg/mL).

**Figure 4:**
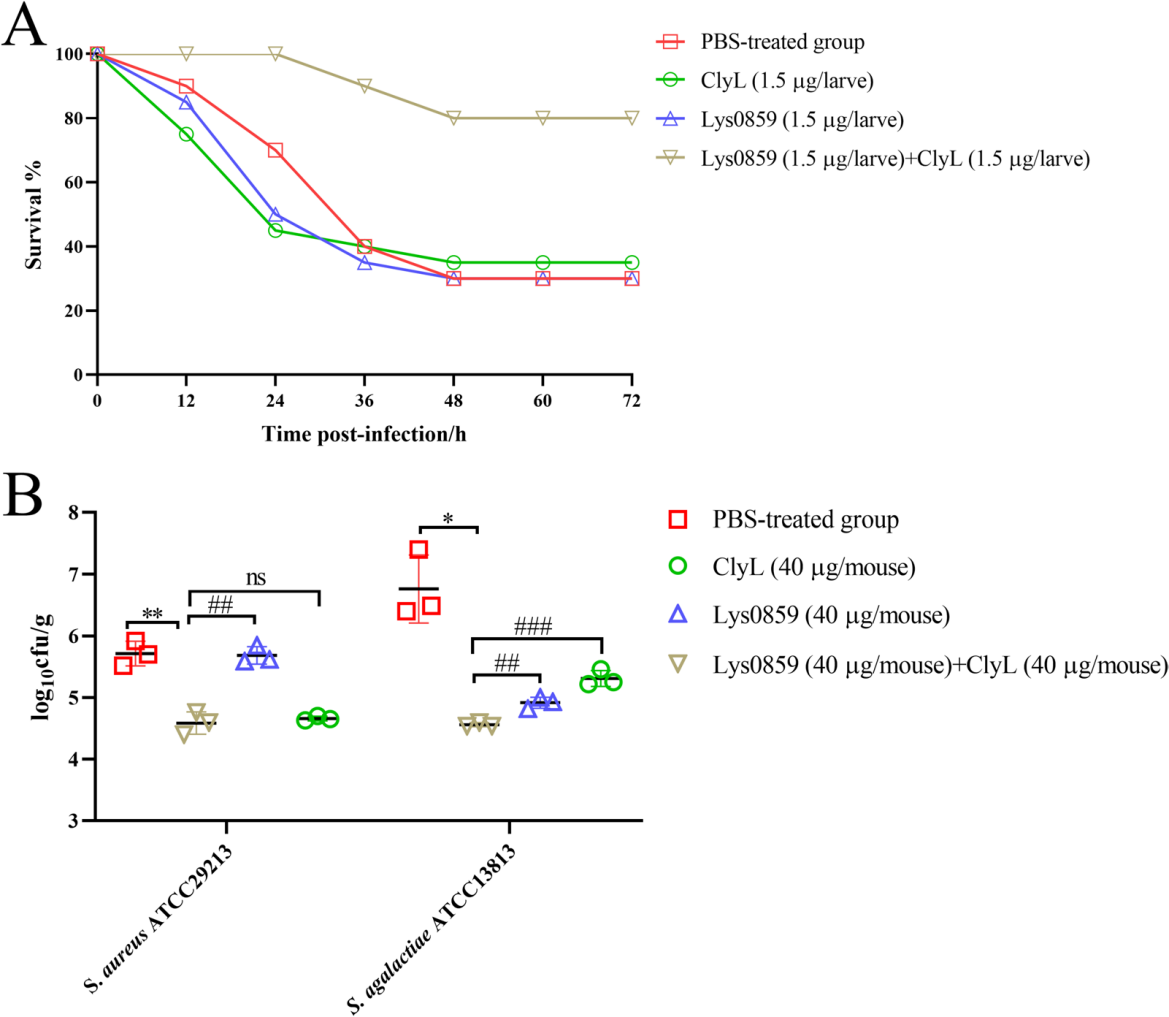
**Effect of phage lysin cocktail treatment on *S. aureus-* and *S. agalactiae*-induced coinfections.** **A** The therapeutic effect of the phage lysin cocktail on *G. mellonella* larvae coinfected with *S. aureus* ATCC29213 and *S. agalactiae* ATCC13813. The larvae were infected with a mixture of *S. aureus* ATCC29213 and *S. agalactiae* ATCC13813 and then treated with a phage lysin cocktail, chimeric lysin ClyL, or phage lysin Lys0859 at 3 h post infection. **B** The therapeutic effect of the phage lysin cocktail on *S. aureus* ATCC29213- and *S. agalactiae* ATCC13813-induced mastitis in mice. The mice were treated with phage lysin cocktail, chimeric lysin ClyL, or phage lysin Lys0859 at 24 h post infection. Significant differences between the PBS groups and the ClyL + Lys0859 groups were determined by Student’s *t* test (* *P* < 0.05 and ** *P* < 0.01). Significant differences between the ClyL + Lys0859 groups and the ClyL and Lys0859 groups were determined via Student’s *t* test (ns *P* > 0.05, ## *P* < 0.01, and ### *P* < 0.001). The error bars represent the standard deviations (SD).

At present, we have demonstrated that ClyL and Lys0859 display the highest levels of activity against *Staphylococcus* and *Streptococcus* strains, resulting in a synergistic bactericidal effect. Numerous studies indicate that bovine mastitis is attributed to multiple bacterial infections [45]. In bovine mastitis, *S. agalactiae* and *S. aureus* play major roles in the formation of biofilms and implantation in the mammary gland [46, 47]. The models of dual-species biofilms, *Galleria mellonella* larvae, and mastitis in mice were used to evaluate the synergistic effect against coinfection with *Staphylococcus* and *Streptococcus* in vitro and in vivo. First, we determined that ClyL and Lys0859 could clear *S. aureus* ATCC29213 and *S. agalactiae* ATCC13813 biofilms (Additional file 11). Thereafter, we constructed a dual-species biofilm model. The biomass and bacterial count of the dual-species biofilms were greatest when 1 × 10^5^ cfu/mL *S. aureus* ATCC29213 or *S. agalactiae* ATCC13813 was cultured in 50% TSB (TSB: PBS = 1:1) for 24 h (Figure 3A–C). The dual-species biofilms of *S. aureus* ATCC29213 and *S. agalactiae* ATCC13813 were subsequently cultured at 37 °C for 24 h and 48 h. Our results indicated that a combination of ClyL (100 μg/mL) and Lys0859 (100 μg/mL) significantly decreased the biomass and bacterial number of dual-species biofilms compared with those in the PBS groups (*P* < 0.01) (Figure 3D and E). The combination of ClyL (100 μg/mL) and Lys0859 (100 μg/mL) or ClyL (50 μg/mL) and Lys0859 (50 μg/mL) was superior to ClyL (100 μg/mL) or Lys0859 (100 μg/mL) alone for clearing biofilms (Figure 3D and E). Moreover, there was no significant difference in antibiofilm ability between ClyL (100 μg/mL) + Lys0859 (100 μg/mL) and ClyL (50 μg/mL) + Lys0859 (50 μg/mL) (Figure 3D and E). The FESEM images of the dual-species biofilms were also consistent with the above results (Figure 3F). To explore the therapeutic effects of a combination of ClyL and Lys0859 in vivo, we developed a coinfection model using *G. mellonella* larvae infected with *S. aureus* ATCC29213 and *S. agalactiae* ATCC13813, as well as a mouse mastitis model. The survival rates of larvae treated with a combination of ClyL (1.5 μg/larvae) and Lys0859 (1.5 μg/larvae), ClyL (1.5 μg/larvae), Lys0859 (1.5 μg/larvae), and PBS were 80%, 35%, 30%, and 30%, respectively (Figure 4A). Previous work has confirmed that intramammary injection of Lys0859 (20 μg/gland) reduces the bacterial burden in mammary tissue by 3.74 logs [27]. In this study, the *S. aureus* and *S. agalactiae* numbers in mammary gland tissue were significantly lower in the ClyL (40 μg/mouse) and Lys0859 (40 μg/mouse) groups than in the PBS groups (*P* < 0.05) (Figure 4B). The combination of ClyL and Lys0859 was superior to the combination of ClyL or Lys0859 alone in eradicating bacteria from *S. aureus* ATCC29213-*S. agalactiae* ATCC13813-coinfected mammary gland tissue (Figure 4B). Together, the combined use of ClyL and Lys0859 may represent a viable approach for addressing coinfections caused by *Staphylococcus* and *Streptococcus*.

### The combination of phage lysin cocktail and cefquinome has a synergistic bactericidal effect in vitro

**Figure 5:**
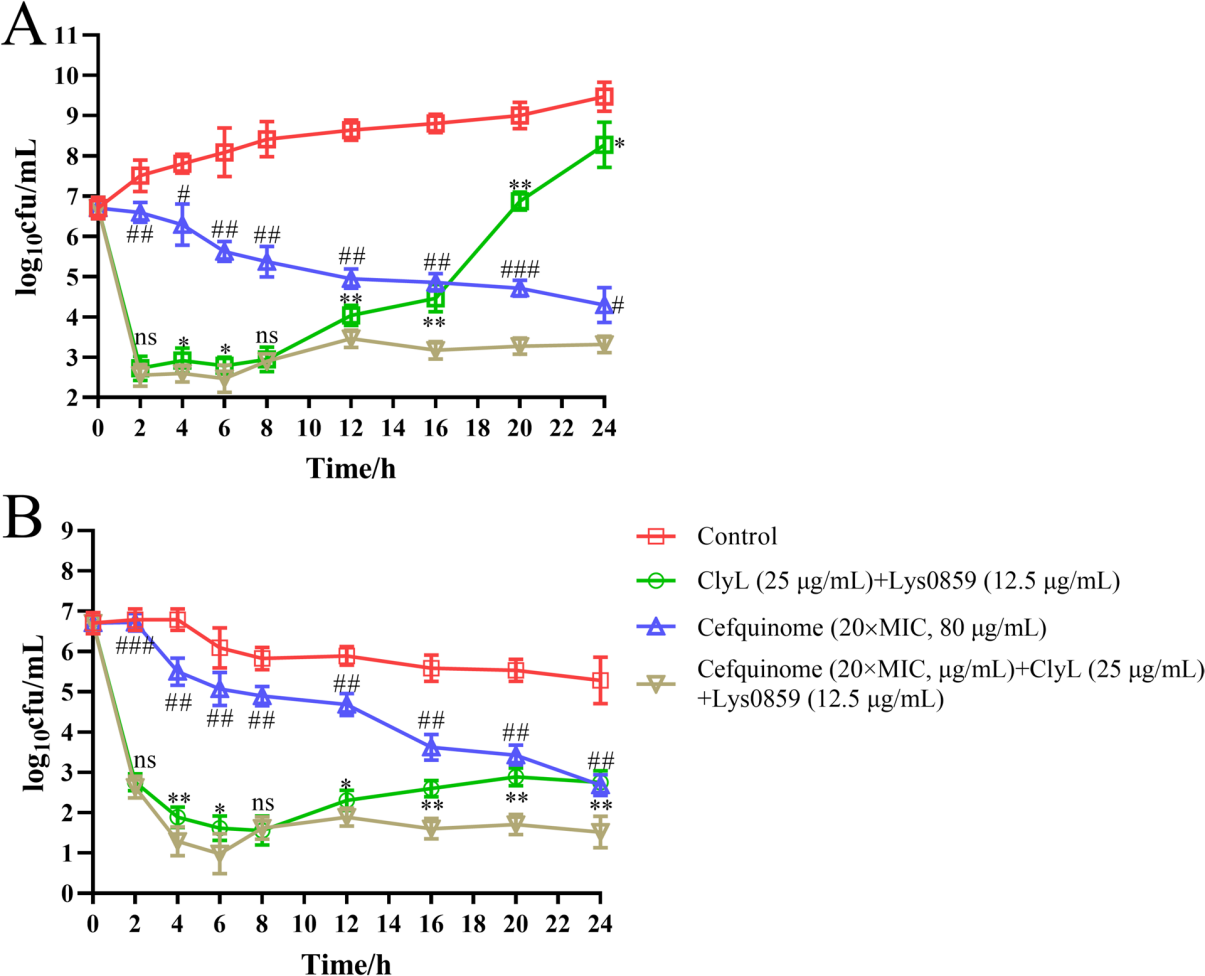
**The antibacterial activity of the combination of phage lysin cocktail and cefquinome in culture medium and semen.** The antibacterial activities of the phage lysin cocktail alone, cefquinome alone, and the combination of cefquinome and phage lysin cocktail were analysed in culture medium (**A**) and semen (**B**). PBS was used as a negative control. *Staphylococcus* 65 was added to the culture medium and semen. The bacterial strains of *Staphylococcus* 65 in the culture medium and semen were incubated at 37 °C for 24 h and tested by plating on TSB agar at different times. Significant differences between the ClyL + Lys0859 groups and the Cefquinome + ClyL + Lys0859 groups were determined via Student’s *t* test (ns *P* > 0.05, * *P* < 0.05, and ** *P* < 0.01). Significant differences between the cefquinome groups and the cefquinome + ClyL + Lys0859 groups were determined by Student’s *t* test (# *P* < 0.05, ## *P* < 0.01, and ### *P* < 0.001). All the experiments were repeated three times. The error bars represent the standard deviation (SD).

Notably, antibiotics and lysin have been theorized to destroy bacteria in distinct ways [48, 49], and a combination of antibiotics and lysin is beneficial for reducing the occurrence of bacterial resistance during treatment [26]. Unexpectedly, in this study, a combination of ClyL and Lys0859 swiftly lowered the bacterial count within 0–8 h but did not affect bacterial growth within 8–24 h (Figure 5A and B). In contrast, cefquinome consistently decreased the visible number of *Staphylococcus* 65 colonies within 24 h, although more slowly than ClyL and Lys0859 did (Figure 5A and B). Our previous study corroborated that the combination of the chimeric lysin ClyQ and mupirocin had a synergistic bactericidal effect on *S. aureus* strains [26]. Therefore, the ClyL + Lys0859, cefquinome alone, and cefquinome + ClyL + Lys0859 groups were designed to explore the synergistic effect of the phage lysin cocktail and cefquinome on bacterial endometritis. As depicted in Figure 5, the combination of phage lysin cocktail and cefquinome in TSB and semen was superior to ClyL + Lys0859 and cefquinome monotherapy in eliminating and inhibiting bacterial proliferation. ClyL and Lys0859 were utilized in the development of a phage lysin cocktail, which was subsequently combined with cefquinome to create a combined therapy for preventing sow endometritis after AI.

### Boar semen containing phage lysin and antibiotics is a viable strategy for preventing bacterial endometritis after AI

**Figure 6:**
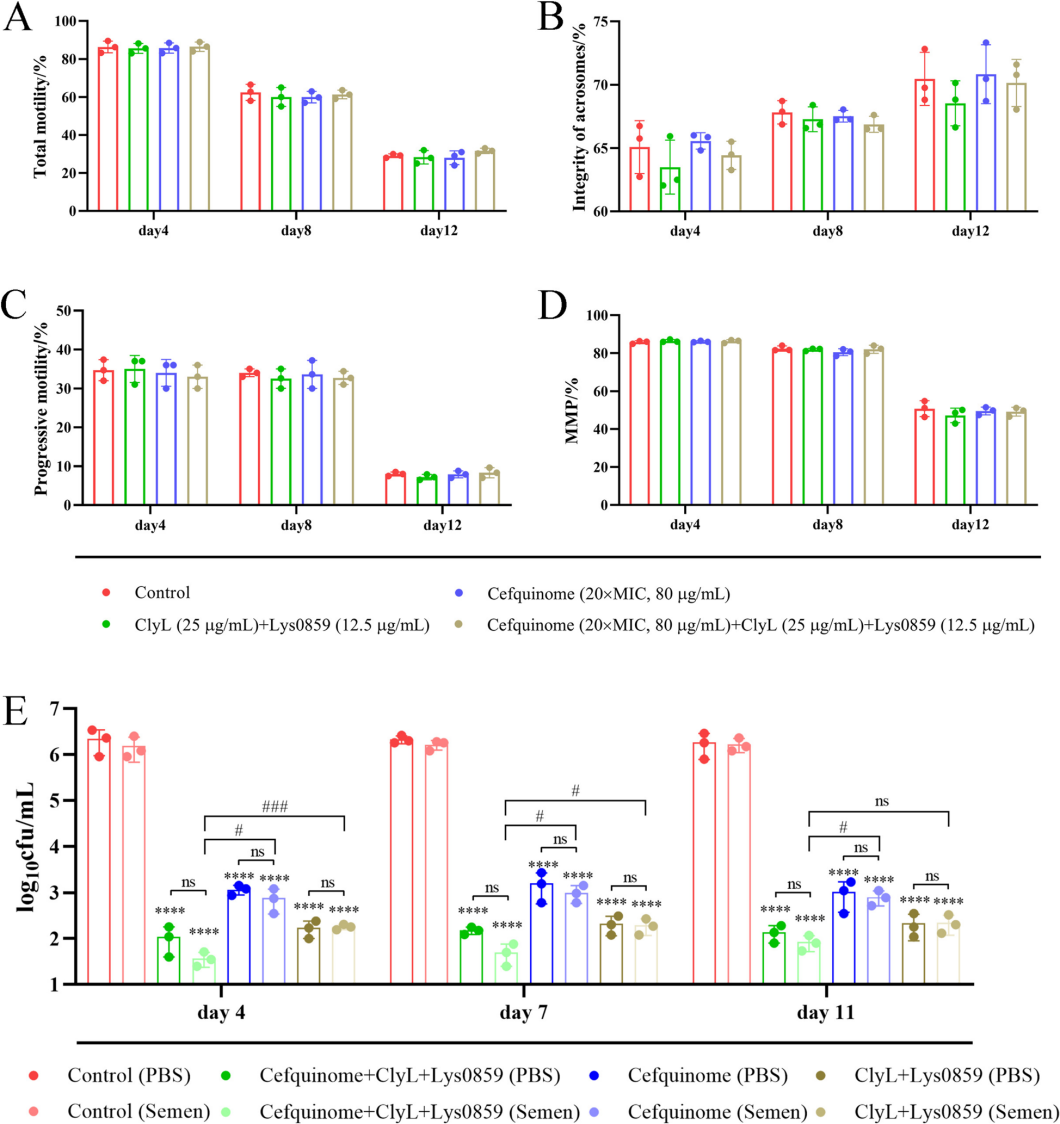
**Effects of ClyL, Lys0859, cefquinome and boar sperm.** **A**–**D** Effects of ClyL, Lys0859, and cefquinome on the activity of boar sperm. The concentrations of ClyL and Lys0859 were 25 and 12.5 μg/mL, respectively. The cefquinome concentration was 20 × MIC (80 μg/mL). PBS, cefquinome, ClyL + Lys0859, and cefquinome + ClyL + Lys0859 were added to boar semen. The semen was subsequently stored at 17 °C, and the total motility (**A**), acrosomal integrity (**B**), progressive motility (**C**), and mitochondrial membrane potential (MMP) (**D**) of the sperm were monitored on the 4^th^, 8^th^, and 12^th^ days of storage. (**E**) boar semen did not significantly affect the antibacterial activity of ClyL, Lys0859, or cefquinome. The PBS, cefquinome, ClyL + Lys0859, and cefquinome + ClyL + Lys0859 were mixed with PBS and boar semen. The mixtures were then stored at 17 °C, and the lytic activities of PBS, cefquinome, ClyL + Lys0859, and cefquinome + ClyL + Lys0859 in PBS and semen against *Staphylococcus* 65 were monitored on the 4^th^, 7^th^, and 11^th^ days of storage. The ClyL + Lys0859, cefquinome, cefquinome + ClyL + Lys0859 and PBS groups were compared via Student’s *t* test (**** *P* < 0.0001). The ClyL + Lys0859, cefquinome and cefquinome + ClyL + Lys0859 groups were compared via Student’s *t* test (ns *P* > 0.05, # *P* < 0.05, ### *P* < 0.001). The error bars represent the standard deviation (SD).

Because bacteria often infect boar semen during collection, processing, and storage and boar semen infected with bacteria can result in sow endometritis AI [50, 51], boar semen containing a phage lysin cocktail and cefquinome was used to control bacteria from semen and prevent sow endometritis via AI in this study. Next, the effects of phage lysin and cefquinome on the activity of semen were analysed, and the results indicated that, compared with those of the control group, the activities (including motility, acrosomal integrity, progressive motility, and mitochondrial membrane potential (MMP)) of semen were not significantly affected by cefquinome, the lysins ClyL and Lys0859 (Figure 6A–D, Additional file 12). Furthermore, compared with PBS, semen did not significantly influence the antibacterial activity of cefquinome, ClyL, or Lys0859 (Figure 6E). Compared with the control conditions, the combination of cefquinome, ClyL, and Lys0859 significantly reduced the number of bacteria in PBS and boar semen (Figure 6E). Importantly, the combination of cefquinome, ClyL, and Lys0859 significantly reduced the bacterial number in boar semen compared with that in the ClyL + Lys0859 and cefquinome alone groups (Figure 6E).

### Combination therapy with phage lysin cocktail and cefquinome reduces the incidence rate of endometritis

Our results revealed that the incidence rate of sow endometritis was 0% (0/7) when the combination of a lysin cocktail and cefquinome was used compared with 50% (3/6) when PBS or cefquinome alone was administered (Table 2). In addition, the incidence rate of sow endometritis was 57.14% (4/7) when the phage lysin cocktail alone was administered. The clinical pregnancy and live birth rates of the PBS-treated group were 85.71% (6/7), and those of the combination-treated group were 100% (7/7) (Table 2). The average cure rates of lysostaphin and oxytetracycline for sow endometritis are 82.5% and 72.17%, respectively [3]. The results revealed that the combination of a phage lysin cocktail and antibiotics is a promising strategy for preventing endometritis in sows. Moreover, one-third of infertile women with RIF suffer from CE caused by common bacteria (*Streptococcus*, *Staphylococcus*, *E. coli*, and *Enterococcus faecalis*), and the success rate of embryo transfer in infertile couples can be influenced by RIF [52–55]. Thus, our findings demonstrated that combined therapy with a phage lysin cocktail and antibiotics can be applied for the treatment of women endometritis in future clinical practice.

**Table 2:** **Reproductive outcomes of sows with endometritis in the PBS, ClyL + Lys0859, cefquinome, and cefquinome + ClyL + Lys0859 groups**

Groups	Clinical pregnancy rate/%	Live birth rate/%	Incidence rate of endometritis/%
Control (*n* = 7)	85.71 (6/7)	85.71 (6/7)	50 (3/6)
ClyL + Lys0859 (*n* = 7)	100 (7/7)	100 (7/7)	57.14 (4/7)
Cefquinome (*n* = 7)	85.71 (6/7)	85.71 (6/7)	50 (3/6)
ClyL + Lys0859 + CEFQUINOME (*n* = 7)	100 (7/7)	100 (7/7)	0 (0/7)

## Discussion

The results of this study suggest that *E. coli*, *Staphylococcus*, and *Streptococcus* (*S. suis*) play pivotal roles in endometritis. Moreover, antibiotics exhibit wide-ranging antibacterial properties, and phage lysin has been shown to rapidly lyse *Staphylococcus* and *S. suis*, with the exception of *E. coli* [15, 24]. Combined therapy with phage lysin and antibiotics delays the development of antibiotic resistance [26]. Hence, we developed a novel therapeutic approach that integrates and leverages the benefits of antibiotics and phage lysins for the treatment of bacterium-induced endometritis. Moreover, most cases of endometritis are polymicrobial and involve aerobic and anaerobic bacteria. Research has shown that obligate anaerobes, including *Peptoniphilus*, *Bacteroides*, and *Clostridium*, are associated with dairy cattle endometritis [56]. The isolation procedure did not consider anaerobic cultivation conditions, which resulted in a lack of information on the obligate anaerobes. In this study, MIC_50_ values were also used to choose the ideal antibiotic owing to the paucity of existing criteria for antibiotics. On the basis of the results of antibiotic resistance, the MIC_50_ values of cefquinome for *Staphylococcus*, *S. suis*, and *E. coli* were 1, 0.06, and 0.12 μg/mL, respectively (Additional files 5, 6, 7). In addition, the MIC_90_ value of cefquinome for both *Staphylococcus* and *S. suis* was 2 μg/mL, which was the lowest among all antibiotics (Additional files 5 and 6). Hence, cefquinome was the most suitable antibiotic against bacteria causing sow endometritis in this study. However, cefquinome, classified as a fourth-generation cephalosporin, is considered a critically important antibiotic for human health according to the World Health Organization (WHO) [57]. Enrofloxacin, an FDA- and China-approved fluoroquinolone, has been used broadly for the treatment of swine disease caused by Gram-positive and Gram-negative bacteria [58, 59]. In this study, the MIC_50_ values of enrofloxacin for *Staphylococcus*, *S. suis*, and *E. coli* were 1, 0.25, and 1 μg/mL, respectively (Additional files 5, 6, 7). Therefore, enrofloxacin may be a more suitable antibiotic for treating sow endometritis than cefquinome in the future.

Thus, it is imperative to exploit phage lysin, which can efficiently lyse both *Staphylococcus* and *S. suis*, for the successful implementation of the proposed combined therapy. Our results implied that ClyL and Lys0859 could effectively lyse and exhibited synergistic activity against *Staphylococcus* and *Streptococcus* strains in sows with endometritis and bovine mastitis. An earlier study demonstrated that phage lysin and cefquinome can treat bacterial infections in swine [60, 61] but not sow endometritis. This study represents the first report of the use of a combination of phage lysin and antibiotic treatment to address sow endometritis, with no observed significant impact on semen quality. However, combination therapy does not eliminate the use of antibiotics. *E. coli*, *Staphylococcus*, and *S. suis* play vital roles in endometritis in this study. ClyL and Lys0859 can lyse *Staphylococcus* and *S. suis*, with the exception of *E. coli*. There have been reports on the use of the *E. coli* phages lysin PlyE146 and Lysep3, which display antimicrobial activity towards *E. coli* [62, 63]. Thus, we can develop a phage lysin that targets *E. coli* to eliminate the use of cefquinome. Taken together, the results revealed that semen containing phage lysin and antibiotics was a viable strategy for preventing bacterial endometritis.

Research has shown that antibiotic therapy can be used for the treatment of endometritis in both animals and women [10, 64]. Although antibiotic therapy resulted in chronic endometritis resolution in 82.3% of patients, 17.6% of patients experienced persistent endometritis [65]. These challenges highlight the importance of diverse antimicrobial strategies. At present, phage lysin has been successfully applied for the treatment of various bacterial infections [31, 66]. The incidence rate of sow endometritis was 57.14% (4/7) when the phage lysin cocktail alone was administered. The clinical pregnancy and live birth rates of the PBS-treated group were 85.71% (6/7), and those of the combination-treated group were 100% (7/7). In accordance with the findings presented above, to address the limitations of antibiotic monotherapy, we developed a potential strategy in which semen was employed as a carrier to deliver a combination of phage lysin cocktail and cefquinome through AI.

In brief, 526 strains consisting of *Staphylococcus* spp. (26.3%, 141/536), *Streptococcus* spp. (12.3%, 66/536), *E. coli* (28.9%, 155/536), *Enterococcus* spp. (20.1%, 108/536), *Proteus* spp. (9.5%, 51/536), and *Corynebacterium* spp. (2.8%, 15/536) were isolated from sow endometritis samples. According to the findings of antibiotic susceptibility testing, *Staphylococcus*, *Streptococcus*, and *E. coli* strains isolated from sow endometritis samples demonstrated sensitivity to cefquinome. A novel antimicrobial agent, the chimeric lysin ClyL, was subsequently developed and demonstrated remarkable bactericidal efficacy against *Staphylococcus* strains obtained from cases of sow endometritis and bovine mastitis. Moreover, the phage lysin Lys0859 had significant effects on *Streptococcus strains* isolated from sows with endometritis and bovine mastitis. ClyL and Lys0859 were used to design a phage lysin cocktail that exhibited synergistic effects against coinfection with *Staphylococcus* and *Streptococcus* in vitro and in vivo. Thus, we conceived a novel therapy that combines cefquinome and phage lysin cocktails containing ClyL and Lys0859 against bacterium-induced sow endometritis. The combination of cefquinome and the phage lysin cocktail did not affect sperm activity but had a synergistic bactericidal effect on semen. Additionally, semen did not significantly influence the bactericidal activity of the phage lysin cocktail combined with cefquinome. Furthermore, this novel approach reduces the incidence rate of sow endometritis by using semen as a vector to deliver a phage lysin cocktail and cefquinome via AI. In conclusion, we speculate that this novel strategy could be applied to prevent endometritis after AI and coinfections with *Staphylococcus* and *Streptococcus*.

## Supplementary Information

**Additional file 1. Primers used to distinguish bacteria from sow endometritis.**

**Additional file 2. Primers used to identify the serotypes of *S. suis*.**

**Additional file 3. Criteria used to determine the antibiotic sensitivity of bacteria from sows with endometritis.**

**Additional file 4. Primers used to construct the chimeric lysin ClyL.**

**Additional file 5. MIC distributions of fourteen antibiotics against *Staphylococcus* spp. (*n* = 141).**

**Additional file 6. MIC distributions of fourteen antibiotics to *S. suis* (*n* = 66).**

**Additional file 7. MIC distributions of eleven antibiotics against *Escherichia coli* (*n* = 155).**

**Additional file 8. Turbidity decreases in the effects of the phage lysins ClyL and Lys0859 against *S. suis*.**

**Additional file 9. Turbidity decrease caused by the phage lysins ClyL and Lys0859 against *Staphylococcus*.**

**Additional file 10. FIC indices for the combinations of ClyL and Lys0859.**

**Additional file 11. Chimeric lysin ClyL and parental phage lysin Lys0859-treated 24-h- and 48-h-old biofilms formed from single *S. aureus* ATCC29213 and *S. agalactiae* ATCC13813 strains.** The biomass of single* S. aureus* ATCC29213 (A) and *S. agalactiae* ATCC13813 (B) biofilms was treated with ClyL and Lys0859 at 37 °C for 2 h. The viable cell counts of single* S. aureus* ATCC29213 (C) and *S. agalactiae* ATCC13813 (D) biofilms were determined after treatment with ClyL and Lys0859 at 37 °C for 2 h. Significant differences between the PBS groups and the ClyL and Lys0859 groups were determined by Student’s *t* test (ns *p* > 0.05, * *p* < 0.05 ** *p* < 0.01, and *** *p* < 0.001). The experiments were performed three times. The data are shown as the mean ± SD.

**Additional file 12. Effects of ClyL, Lys0859 and cefquinome on various kinematic parameters (motility) of sperm.**

## Data Availability

The datasets used and analysed during the current study are available from the corresponding author upon reasonable request.
