# Supplementary material for: Development of a novel chimeric lysin to combine parental phage lysin and cefquinome for preventing sow endometritis after artificial insemination
Source: Vet Res. 2025 Feb 11;56:39. doi: 10.1186/s13567-025-01457-4 (PMC11816537; doi:10.1186/s13567-025-01457-4)
Supplement: Supplementary file 1 — Additional file 1. Primers used to distinguish bacteria from sow endometritis. [file 13567_2025_1457_MOESM1_ESM.doc]

**Additional file 1 The primers were used to identify the bacteria from sow endometritis.**

| Genes | Primers sequences | Target fragment length (bp) |
| --- | --- | --- |
| *Escherichia coli*-phoA | Forward: 5’-CGATTCTGGAAATGGCAAAAG-3’ | 720 |
|  | Reverse: 5’-CGTGATCAGCGGTGACTATGAC-3’ |  |
| *Staphylococcus*-tuf | Forward: 5’-TACCAGCATTAGTAGTATTCTTAAACAAAGTTG-3’ | 143 |
|  | Reverse: 5’- TGCTGAACCAGCGATTACAG-3’ |  |
| *Streptococcus suis*-gdh | Forward: 5’- GCAGCGTATTCGTCAAACG-3’ | 688 |
|  | Reverse: 5’- CCATGGACGATAAAGATGG-3’ |  |
| *Streptococcus suis*-recN | Forward: 5’- CTACAAACAGCTCTCTTCT-3’ | 336 |
|  | Reverse: 5’- ACAACAGCCAATTCATGGCGTGATT-3’ |  |
| *Enterococcus*-ent | Forward: 5’- TACTGACAAACCATTCATGATG-3’ | 112 |
|  | Reverse: 5’- AATTCGTCACCAACGCGAAC-3’ |  |
| *Proteus*-tuf | Forward: 5’- AAATTGTTGAATTAGCAGAAGCA-3’ | 541 |
|  | Reverse: 5’- GCGATTGGGTGGATCAGTTC-3’ |  |
| *Corynebacterium*-rpoB | Forward: 5’- CGTATGAACATCGGCCAGGT-3’ | 446 |
|  | Reverse: 5’- TCCATTTCGCCGAAGCGCTG-3’ |  |
| Bacterium-16SrRNA | Forward: 5’- AGAGTTTGATCCTGGCTCAG-3’ | 1492 |
|  | Reverse: 5’- GGTTACCTTGTTACGACTT-3’ |  |
