# Supplementary material for: Development of a novel chimeric lysin to combine parental phage lysin and cefquinome for preventing sow endometritis after artificial insemination
Source: Vet Res. 2025 Feb 11;56:39. doi: 10.1186/s13567-025-01457-4 (PMC11816537; doi:10.1186/s13567-025-01457-4)
Supplement: Supplementary file 2 — Additional file 2. Primers used to identify the serotypes of S. suis. [file 13567_2025_1457_MOESM2_ESM.doc]

**Additional file 2 The primers were used to identify the serotypes of *S. suis*.**

| Cps group | Cps type | Primers sequences | Target fragment length (bp) |
| --- | --- | --- | --- |
| Ⅰ | 3 | Forward: 5’-GGTTTTGATTGGTCTAGTTG-3’ | 214 |
|  |  | Reverse: 5’-CTCTAAAGCTCGATATCTAC-3’ |  |
|  | 13 | Forward: 5’-TATGGTTAAAGGTGGAACTG-3’ | 408 |
|  |  | Reverse: 5’-CCTTGTATATATTCCCTCCA-3’ |  |
|  | 18 | Forward: 5’-TAATGGGATAGTTGCGTTAC-3’ | 617 |
|  |  | Reverse: 5’-ATACATAAAGTTGTCCTGCG-3’ |  |
| Ⅱ | 2 and 1/2 | Forward: 5’-TTAGCAACGTTGCCAATAAG-3’ | 173 |
|  |  | Reverse: 5’-AATCCTCCATTAAAACCCTG-3’ |  |
|  | 6 | Forward: 5’-GCTCACTATTTTTACATTACAC-3’ | 278 |
|  |  | Reverse: 5’-TATTACTCCGCCAAATACAG-3’ |  |
|  | 1 and 14 | Forward: 5’-TTAGACAGACACCTTATAGG-3’ | 386 |
|  |  | Reverse: 5’-CTAGCTTCGTTACTTGATTC-3’ |  |
|  | 16 | Forward: 5’-AAGGTTATCCACGAAAGATG-3’ | 494 |
|  |  | Reverse: 5’-TCCGGCAATATTCTTTCAAG-3’ |  |
|  | 27 | Forward: 5’-AGACACTGCTTGCATTATTG-3’ | 655 |
|  |  | Reverse: 5’-TCAGAATTACTTCCTGTTGC-3’ |  |
| Ⅲ | 21 | Forward: 5’-TATCATATTGAGAATCTTCCC-3’ | 160 |
|  |  | Reverse : 5’-TTGCGTAGCATACAAAGTTC-3’ |  |
|  | 28 | Forward: 5’-ATTATGTTGGTTGCAGAAGG-3’ | 272 |
|  |  | Reverse: 5’-CGACTCAATTGTTGTAGTAG-3’ |  |
|  | 29 | Forward: 5’-TTCTGGGATTTTAGGAATGC-3’ | 415 |
|  |  | Reverse: 5’-CATGAAATACGCACTTGTAC-3’ |  |
|  | 30 | Forward: 5’-TATTGCACTAGCTTCAGAAC-3’ | 568 |
|  |  | Reverse: 5’-TGCATCCATAGTTGTATTCG-3’ |  |
| Ⅳ | 4 | Forward: 5’-GACTATCTGTATACCCAAAC-3’ | 903 |
|  |  | Reverse: 5’-TCCTTCCAAGTATTCTCTAG-3’ |  |
|  | 5 | Forward: 5’-ATCTTAGGAATGATTCGGAC-3’ | 720 |
|  |  | Reverse: 5’-ACCAGATATCTGAGCAAATG-3’ |  |
|  | 7 | Forward: 5’-AACTACCTACCTGAACTTTG-3’ | 566 |
|  |  | Reverse: 5’-AGTCTAAAAGTGATCGAGTC-3’ |  |
|  | 17 | Forward: 5’-TAGCATCAGTTTATACGAGG-3’ | 455 |
|  |  | Reverse: 5’-TAGTTTATCTGTGACACACC-3’ |  |
|  | 19 | Forward: 5’-GTGTCGCAAATCAAGTATTG-3’ | 348 |
|  |  | Reverse: 5’-AAGCTAGTACAACAAGCATG-3’ |  |
|  | 23 | Forward: 5’-TAATGTATGCTCTGTCACTG-3’ | 221 |
|  |  | Reverse: 5’-AACGAAACGGAATAGTTTGC-3’ |  |
| Ⅴ | 8 | Forward: 5’-AAATAAGGTAGGAGCTACTC-3’ | 446 |
|  |  | Reverse: 5’-ATCCAACCTTAGCTTTCTGT-3’ |  |
|  | 15 | Forward: 5’-ATCGTTTTGAGATTGAGTGG-3’ | 542 |
|  |  | Reverse: 5’-TAAACGGATTCGGTTACTCA-3’ |  |
|  | 25 | Forward: 5’-GTTTGCTCCGATCATAATAG-3’ | 174 |
|  |  | Reverse: 5’-CCAGTAAAAGGACTCAATAC-3’ |  |
| Ⅵ | 9 | Forward: 5’-GAAAGTAGGTATATCTCAGC-3’ | 368 |
|  |  | Reverse: 5’-GGGCTATTAAAACTCCTATC-3’ |  |
|  | 10 | Forward: 5’-TTTCCCATTTGCTTATGGAC-3’ | 633 |
|  |  | Reverse: 5’-GGAATAAAAACGATTGGGAG-3’ |  |
|  | 11 | Forward: 5’-ATGCGATTGCAACAATTGAC-3’ | 833 |
|  |  | Reverse: 5’-AGGCATGAGTAATACATAGG-3’ |  |
|  | 12 | Forward: 5’-AACAGGTATTTCAGGATTGC-3’ | 131 |
|  |  | Reverse: 5’-CTCGGATAAAGATAATCAGC-3’ |  |
|  | 24 | Forward: 5’-TACTGAGATTTATTGGGACG-3’ | 224 |
|  |  | Reverse: 5’-AAGCGATTGGATTACATTGC-3’ |  |
| Ⅶ | 31 | Forward: 5’-ACAATCGTTTCTGCAATACG-3’ | 842 |
|  |  | Reverse: 5’-GATGAAAACATCGTTGGTAG-3’ |  |
|  |  | Forward: 5’-ATCAGTAGTGGGAATAGTTG-3’ | 423 |
|  |  | Reverse: 5’-TTTACTGTTTTTCGACCGTG-3’ |  |
| Cps: capsular polysaccharide | | | |
