# Supplementary material for: Development of a novel chimeric lysin to combine parental phage lysin and cefquinome for preventing sow endometritis after artificial insemination
Source: Vet Res. 2025 Feb 11;56:39. doi: 10.1186/s13567-025-01457-4 (PMC11816537; doi:10.1186/s13567-025-01457-4)
Supplement: Supplementary file 3 — Additional file 3. Criteria used to determine the antibiotic sensitivity of bacteria from sows with endometritis. [file 13567_2025_1457_MOESM3_ESM.doc]

**Additional file 3 The criteria were used to determine the antibiotic sensitivity of bacteria from sow endometritis.**

| Antibiotics | *Staphylococcus* | | |  | *Streptococcus suis* | | |  | *Escherichia coli* | | |
| --- | --- | --- | --- | --- | --- | --- | --- | --- | --- | --- | --- |
|  | Breakpoints (μg/mL) | | |  | Breakpoints (μg/mL) | | |  | Breakpoints (μg/mL) | | |
|  | S | I | R |  | S | I | R |  | S | I | R |
| Ampicillin | - | - | - |  | ≤0.5 | 1 | ≥2 |  | ≤8 | 16 | ≥32 |
| Amoxicillin | - | - | - |  | ≤2 | 4 | ≥8 |  | - | - | - |
| Penicillin G | ≤0.12 | - | ≥0.25 |  | ≤0.25 | 0.5 | ≥1 |  | - | - | - |
| Ceftiofur | ≤2 | 4 | ≥8 |  | ≤2 | 4 | ≥8 |  | ≤2 | 4 | ≥8 |
| Cefquinome | ≤4 | 8 | ≥16 |  | - | - | - |  | - | - | - |
| Gentamicin | - | - | - |  | - | - | - |  | ≤4 | 8 | ≥16 |
| Tetracycline | ≤4 | 8 | ≥16 |  | ≤0.5 | 1 | ≥2 |  | ≤4 | 8 | ≥16 |
| Doxycycline | ≤4 | 8 | ≥16 |  | ≤0.25 | 0.5 | ≥1 |  | ≤4 | 8 | ≥16 |
| Florfenicol | - | - | - |  | ≤2 | 4 | ≥8 |  | ≤4 | 8 | ≥16 |
| Chloramphenicol | ≤8 | 16 | ≥32 |  | ≤4 | 8 | ≥16 |  | ≤8 | 16 | ≥32 |
| Amikacin | - | - | - |  | - | - | - |  | ≤16 | 16 | ≥64 |
| Erythromycin | ≤0.5 | 1-4 | ≥8 |  | ≤0.25 | 0.5 | ≥1 |  | - | - | - |
| Timicosin | - | - | - |  | - | - | - |  | - | - | - |
| Lincomycin | ≤0.5 | 1-2 | ≥4 |  | ≤0.25 | 0.5 | ≥1 |  | - | - | - |
| Enrofloxacin | - | - | - |  | ≤0.25 | 0.5 | ≥1 |  | - | - | - |
