# Supplementary material for: Development of a novel chimeric lysin to combine parental phage lysin and cefquinome for preventing sow endometritis after artificial insemination
Source: Vet Res. 2025 Feb 11;56:39. doi: 10.1186/s13567-025-01457-4 (PMC11816537; doi:10.1186/s13567-025-01457-4)
Supplement: Supplementary file 4 — Additional file 4. Primers used to construct the chimeric lysin ClyL. [file 13567_2025_1457_MOESM4_ESM.doc]

**Additional file 4 The primers were used to construct the chimeric lysin ClyL.**

| Primers | Sequences (5'-3') |
| --- | --- |
| LysGH15CHAP-F | GGATCCATGGCAAAAACACAAGCTGA |
| LysGH15CHAP-R | CGATCCAGACGAGCCTCCGGCTTTAACCGGGATC |
| Lys0859CBD-F | GGAGGCTCGTCTGGATCGATGACAACAGTATTTGAA |
| Lys0859CBD-R | AAGCTTTTATTTGAAAATACCATAAGGC |
| The restriction sites and linker are underlined. *Bam*HI: GGATCC, *Hin*dIII: AAGCTT, linker: GGAGGCTCGTCTGGATCG. | |
