# Supplementary material for: Development of a novel chimeric lysin to combine parental phage lysin and cefquinome for preventing sow endometritis after artificial insemination
Source: Vet Res. 2025 Feb 11;56:39. doi: 10.1186/s13567-025-01457-4 (PMC11816537; doi:10.1186/s13567-025-01457-4)
Supplement: Supplementary file 5 — Additional file 5. MIC distributions of fourteen antibiotics against Staphylococcus spp. (n = 141). [file 13567_2025_1457_MOESM5_ESM.doc]

**Additional file 5 The MICs distribution of fourteen antibiotics to *Staphylococcus* spp. (*n* = 141).**

| Antibiotics | The values of MIC (μg/mL) | | | | | | | | | | | | | | | | | MIC_50_ (μg/mL) | MIC_90_ (μg/mL) |
| --- | --- | --- | --- | --- | --- | --- | --- | --- | --- | --- | --- | --- | --- | --- | --- | --- | --- | --- | --- |
|  | 0.015 | 0.03 | 0.06 | 0.12 | 0.25 | 0.5 | 1 | 2 | 4 | 8 | 16 | 32 | 64 | 128 | 256 | 512 | 1024 |  |  |
| Ampicillin | 3 | 3 | 15 | 12 | 6 | 11 | 5 | 7 | 5 | 11 | 8 | 16 | 39 |  |  |  |  | 8 | ≥64 |
| Amoxicillin |  | 2 | 6 | 16 | 11 | 11 | 8 | 6 | 10 | 7 | 12 | 13 | 39 |  |  |  |  | 8 | ≥64 |
| Penicillin G | 4 | 18 | 3 | 7 | 5 | 6 | 3 | 7 | 6 | 10 | 16 | 16 | 40 |  |  |  |  | 16 | ≥64 |
| Ceftiofur |  |  |  |  | 3 | 25 | 30 | 12 | 25 | 25 | 11 | 4 | 6 |  |  |  |  | 4 | 16 |
| Cefquinome |  |  | 1 |  | 21 | 48 | 27 | 27 | 11 | 3 | 1 | 1 | 1 |  |  |  |  | 1 | 2 |
| Gentamicin | 1 | 6 | 5 | 5 | 13 | 21 | 8 | 9 | 13 | 8 | 8 | 12 | 32 |  |  |  |  | 4 | ≥64 |
| Tetracycline |  | 3 | 6 | 13 | 2 | 3 | 2 | 7 | 49 | 17 | 10 | 13 | 16 |  |  |  |  | 4 | ≥64 |
| Doxycycline | 4 | 11 | 5 | 5 | 1 | 6 | 14 | 22 | 26 | 32 | 10 | 4 | 1 |  |  |  |  | 4 | 16 |
| Florfenicol |  |  |  |  |  |  | 1 | 12 | 10 | 2 | 4 | 43 | 69 |  |  |  |  | 32 | ≥64 |
| Chloramphenicol | |  |  |  |  |  |  | 3 | 16 | 4 | 28 | 49 | 41 |  |  |  |  | 32 | ≥64 |
| Erythromycin |  |  |  | 11 | 4 | 5 |  |  |  | 1 |  | 2 |  |  |  | 1 | 117 | ≥1028 | ≥1028 |
| Timicosin |  |  |  |  |  | 1 | 1 | 7 | 9 | 5 | 2 | 3 | 1 | 1 |  | 1 | 110 | ≥1028 | ≥1028 |
| Lincomycin |  |  |  |  |  | 5 | 7 | 4 |  | 2 | 2 | 1 | 2 | 2 |  |  | 116 | ≥1028 | ≥1028 |
| Enrofloxacin |  | 3 | 22 | 18 | 10 | 17 | 16 | 3 | 30 | 13 | 3 | 5 | 1 |  |  |  |  | 1 | 8 |
