# Supplementary material for: Development of a novel chimeric lysin to combine parental phage lysin and cefquinome for preventing sow endometritis after artificial insemination
Source: Vet Res. 2025 Feb 11;56:39. doi: 10.1186/s13567-025-01457-4 (PMC11816537; doi:10.1186/s13567-025-01457-4)
Supplement: Supplementary file 6 — Additional file 6. MIC distributions of fourteen antibiotics to S. suis (n = 66). [file 13567_2025_1457_MOESM6_ESM.doc]

**Additional file 6 The MICs distribution of fourteen antibiotics to *Streptococcus suis* (*n* = 66).**

| Antibiotics | The values of MIC (μg/mL) | | | | | | | | | | | | | | | | | MIC_50_ (μg/mL) | MIC_90_ (μg/mL) |
| --- | --- | --- | --- | --- | --- | --- | --- | --- | --- | --- | --- | --- | --- | --- | --- | --- | --- | --- | --- |
|  | 0.015 | 0.03 | 0.06 | 0.12 | 0.25 | 0.5 | 1 | 2 | 4 | 8 | 16 | 32 | 64 | 128 | 256 | 512 | 1024 |  |  |
| Ampicillin | 4 | 19 | 28 | 8 | 1 | 1 |  | 2 | 1 | 2 |  |  |  |  |  |  |  | 0.06 | 0.25 |
| Amoxicillin | 12 | 38 | 8 | 2 | 1 |  |  | 2 | 1 | 2 |  |  |  |  |  |  |  | 0.03 | 0.12 |
| Penicillin G | 2 | 2 | 4 | 7 | 6 | 22 | 15 | 4 |  | 3 | 1 |  |  |  |  |  |  | 0.5 | 2 |
| Ceftiofur | 3 | 18 | 15 | 5 | 5 | 1 | 7 | 7 | 4 | 1 |  |  |  |  |  |  |  | 0.06 | 2 |
| Cefquinome | 3 | 16 | 20 | 6 | 3 | 7 | 4 | 5 | 1 | 1 |  |  |  |  |  |  |  | 0.06 | 2 |
| Gentamicin |  |  |  |  |  |  | 1 | 1 | 7 | 24 | 27 | 2 | 2 | 2 |  |  |  | 8 | 16 |
| Tetracycline |  |  |  |  | 1 |  |  |  |  | 2 | 28 | 33 | 1 | 1 |  |  |  | 16 | 32 |
| Doxycycline |  |  |  | 1 |  | 3 | 1 | 16 | 29 | 16 |  |  |  |  |  |  |  | 4 | 8 |
| Florfenicol |  |  |  |  |  | 4 | 17 | 20 | 4 | 8 | 10 | 3 |  |  |  |  |  | 2 | 16 |
| Chloramphenicol | |  |  |  |  |  | 8 | 22 | 24 | 10 | 1 | 1 |  |  |  |  |  | 4 | 8 |
| Erythromycin |  |  |  |  | 2 | 2 | 3 | 6 | 14 | 4 | 2 | 2 | 4 | 2 | 3 | 3 | 19 | 16 | ≥1024 |
| Timicosin |  |  |  |  |  |  |  |  |  |  |  |  | 2 | 3 | 9 | 19 | 33 | 512 | ≥1024 |
| Lincomycin |  |  |  |  |  |  |  |  |  |  |  |  | 1 | 2 | 15 | 46 | 2 | 512 | 512 |
| Enrofloxacin |  |  |  | 9 | 38 | 12 |  | 1 | 1 | 4 | 1 |  |  |  |  |  |  | 0.25 | 2 |
