# Supplementary material for: Development of a novel chimeric lysin to combine parental phage lysin and cefquinome for preventing sow endometritis after artificial insemination
Source: Vet Res. 2025 Feb 11;56:39. doi: 10.1186/s13567-025-01457-4 (PMC11816537; doi:10.1186/s13567-025-01457-4)
Supplement: Supplementary file 7 — Additional file 7. MIC distributions of eleven antibiotics against Escherichia coli (n = 155). [file 13567_2025_1457_MOESM7_ESM.doc]

**Additional file 7 The MICs distribution of eleven antibiotics to *Escherichia coli* (*n* =**155).

| Antibiotics | The values of MIC (μg/mL) | | | | | | | | | | | | | | | | | MIC_50_ (μg/mL) | MIC_90_ (μg/mL) |
| --- | --- | --- | --- | --- | --- | --- | --- | --- | --- | --- | --- | --- | --- | --- | --- | --- | --- | --- | --- |
|  | 0.015 | 0.03 | 0.06 | 0.12 | 0.25 | 0.5 | 1 | 2 | 4 | 8 | 16 | 32 | 64 | 128 | 256 | 512 | 1024 |  |  |
| Ampicillin |  |  |  |  |  |  | 2 | 6 | 17 | 4 |  |  |  | 126 |  |  |  | ≥128 | ≥128 |
| Ceftiofur |  |  |  |  | 15 | 55 | 11 |  | 2 |  | 7 | 2 |  | 63 |  |  |  | 1 | ≥128 |
| Cefquinome |  | 14 | 40 | 31 | 4 | 2 | 4 |  |  |  |  |  |  | 60 |  |  |  | 0.12 | ≥128 |
| Gentamicin |  |  |  |  |  |  | 2 | 9 | 59 | 50 | 16 | 4 | 4 | 11 |  |  |  | 8 | 32 |
| Amikacin |  |  |  |  |  |  |  |  | 4 | 11 | 61 | 66 | 13 |  |  |  |  | 32 | 32 |
| Tetracycline |  |  |  |  |  |  | 22 | 16 | 2 | 4 | 43 | 27 | 16 | 25 |  |  |  | 16 | ≥128 |
| Doxycycline |  |  |  |  |  |  | 13 | 25 | 2 | 18 | 60 | 32 | 5 |  |  |  |  | 16 | 32 |
| Florfenicol |  |  |  |  |  |  |  |  | 7 | 40 | 9 | 4 |  | 95 |  |  |  | ≥128 | ≥128 |
| Chloramphenicol |  |  |  |  |  |  |  |  | 25 | 20 |  |  | 8 | 102 |  |  |  | ≥128 | ≥128 |
| Timicoxin |  |  |  |  |  |  | 8 | 22 | 24 | 10 | 1 | 1 |  |  |  |  |  | 128 | 256 |
| Enrofloxacin |  |  |  |  | 2 | 2 | 3 | 6 | 14 | 4 | 2 | 2 | 4 | 2 | 3 | 3 | 19 | 1 | 64 |
