# Supplementary material for: Development of a novel chimeric lysin to combine parental phage lysin and cefquinome for preventing sow endometritis after artificial insemination
Source: Vet Res. 2025 Feb 11;56:39. doi: 10.1186/s13567-025-01457-4 (PMC11816537; doi:10.1186/s13567-025-01457-4)
Supplement: Supplementary file 8 — Additional file 8. Turbidity decreases in the effects of the phage lysins ClyL and Lys0859 against S. suis. [file 13567_2025_1457_MOESM8_ESM.doc]

***Additional file 8 The turbidity decrease of phage lysin ClyL and Lys0859 against S. suis.***

| Strains | Serotype of strains | Decrease of turbidity/% | | | | | | |
| --- | --- | --- | --- | --- | --- | --- | --- | --- |
|  |  | ClyL | | |  | Lys0859 | | |
| *S. suis* 6 | 16 | 53.95 | 54.70 | 54.42 |  | 86.42 | 86.64 | 86.77 |
| *S. suis* 9 | 19 | 84.02 | 83.46 | 83.09 |  | 88.86 | 88.67 | 89.11 |
| *S. suis* 14 | 29 | 29.15 | 27.13 | 26.46 |  | 71.82 | 71.87 | 72.03 |
| *S. suis* 15 | / | 69.61 | 69.10 | 68.36 |  | 53.36 | 53.60 | 54.23 |
| *S. suis* 19 | 5 | 30.39 | 31.42 | 25.29 |  | 80.95 | 80.79 | 79.27 |
| *S. suis* 29 | 16 | 41.89 | 36.84 | 39.35 |  | 89.14 | 88.12 | 89.06 |
| *S. suis* 30 | 16 | 50.75 | 50.32 | 51.97 |  | 87.84 | 87.98 | 87.97 |
| *S. suis* 31 | 12 | 30.09 | 32.56 | 33.01 |  | 75.12 | 71.71 | 74.50 |
| *S. suis* 33 | 12 | 29.89 | 30.55 | 29.90 |  | 75.21 | 75.16 | 74.32 |
| *S. suis* 34 | 12 | 34.69 | 32.71 | 31.48 |  | 78.71 | 78.87 | 77.78 |
| *S. suis* 36 | / | 52.44 | 54.17 | 53.11 |  | 80.34 | 82.73 | 82.82 |
| *S. suis* 40 | 12 | 32.41 | 33.07 | 33.10 |  | 74.46 | 74.75 | 75.07 |
| *S. suis* 43 | / | 44.38 | 43.31 | 43.72 |  | 83.86 | 83.00 | 83.48 |
| *S. suis* 44 | 28 | 52.33 | 52.43 | 52.63 |  | 81.52 | 81.27 | 81.55 |
| *S. suis* 45 | 5 | 29.25 | 27.89 | 24.82 |  | 78.05 | 78.25 | 77.16 |
| *S. suis* 47 | 12 | 53.94 | 53.15 | 55.25 |  | 85.24 | 85.27 | 85.49 |
| *S. suis* 51 | 12 | 41.44 | 41.38 | 40.76 |  | 81.64 | 81.22 | 81.24 |
| *S. suis* 52 | 15 | 28.15 | 27.13 | 23.46 |  | 72.82 | 72.87 | 72.03 |
| *S. suis* 53 | 12 | 21.44 | 20.98 | 19.68 |  | 77.24 | 76.79 | 76.61 |
| *S. suis* 55 | 12 | 25.39 | 26.15 | 27.02 |  | 77.91 | 78.37 | 79.31 |
| *S. suis* 56 | 12 | 64.48 | 62.99 | 63.85 |  | 73.10 | 72.44 | 72.28 |
| *S. suis* 62 | 5 | 44.95 | 45.62 | 45.27 |  | 82.28 | 82.26 | 82.43 |
| *S. suis* 63 | 16 | 40.56 | 40.97 | 39.77 |  | 89.19 | 89.14 | 89.18 |
| *S. suis* 64 | 16 | 23.27 | 21.75 | 25.43 |  | 69.40 | 69.23 | 70.21 |
| *S. suis* 66 | 12 | 12.57 | 15.10 | 14.89 |  | 64.24 | 64.18 | 64.13 |
| *S. suis* 68 | 12 | 22.45 | 22.51 | 21.52 |  | 69.76 | 69.55 | 70.02 |
| *S. suis* 71 | 5 | 52.09 | 51.41 | 51.51 |  | 87.61 | 87.47 | 87.53 |
| *S. suis* 75 | 12 | 37.97 | 37.83 | 37.20 |  | 77.27 | 76.65 | 77.51 |
| *S. suis* 83 | 9 | 33.07 | 37.86 | 37.44 |  | 75.97 | 76.16 | 76.10 |
| *S. suis* 84 | 12 | 17.79 | 18.24 | 19.66 |  | 69.47 | 69.77 | 70.06 |
| *S. suis* 87 | 12 | 33.90 | 36.49 | 35.19 |  | 76.25 | 76.30 | 76.11 |
| *S. suis* 91 | 15 | 21.81 | 21.29 | 22.03 |  | 70.19 | 69.86 | 70.42 |
| *S. suis* 94 | 5 | 37.68 | 35.77 | 36.31 |  | 80.43 | 79.77 | 79.58 |
| *S. suis* 99 | 12 | 22.86 | 24.52 | 22.23 |  | 77.59 | 77.57 | 76.92 |
| *S. suis* 100 | 12 | 26.40 | 29.87 | 27.53 |  | 80.27 | 80.04 | 80.13 |
| *S. suis* 105 | 12 | 13.88 | 14.02 | 14.87 |  | 62.24 | 62.01 | 62.36 |
| *S. suis* 106 | 9 | 81.30 | 80.97 | 80.67 |  | 83.82 | 83.75 | 83.63 |
| *S. suis* 114 | 12 | 59.90 | 59.99 | 59.90 |  | 84.10 | 83.97 | 83.67 |
| *S. suis* 121 | 12 | 42.81 | 43.16 | 42.93 |  | 78.99 | 78.93 | 78.65 |
| *S. suis* 133 | 30 | 30.66 | 29.77 | 30.75 |  | 74.94 | 74.63 | 69.05 |
| *S. suis* 134 | 12 | 62.64 | 62.55 | 62.59 |  | 82.98 | 83.16 | 83.33 |
| *S. suis* 136 | 16 | 56.38 | 56.54 | 56.62 |  | 67.45 | 66.50 | 66.71 |
| *S. suis* 137 | 16 | 41.35 | 41.90 | 41.09 |  | 72.44 | 70.51 | 72.33 |
| *S. suis* 139 | 28 | 40.76 | 41.56 | 41.08 |  | 76.40 | 76.89 | 77.18 |
| *S. suis* 146 | 12 | 59.59 | 59.89 | 60.68 |  | 81.83 | 81.98 | 82.19 |
| *S. suis* 147 | 31 | 74.03 | 73.93 | 72.54 |  | 76.94 | 77.01 | 76.02 |
| *S. suis* 151 | 12 | 56.26 | 55.37 | 56.06 |  | 81.31 | 81.63 | 82.14 |
| *S. suis* 153 | 12 | 27.54 | 27.50 | 27.71 |  | 76.00 | 76.32 | 76.53 |
| *S. suis* 154 | 12 | 49.71 | 49.44 | 49.96 |  | 80.09 | 79.93 | 79.68 |
| *S. suis* 155 | 12 | 53.38 | 51.99 | 53.37 |  | 82.68 | 82.20 | 82.82 |
| *S. suis* 156 | 25 | 61.26 | 61.58 | 61.65 |  | 77.20 | 79.81 | 79.58 |
| *S. suis* 161 | 12 | 39.70 | 40.77 | 41.06 |  | 81.33 | 81.20 | 81.10 |
| *S. suis* 163 | 5 | 33.00 | 29.90 | 32.02 |  | 86.12 | 85.86 | 85.80 |
| *S. suis* 164 | 5 | 36.50 | 35.98 | 36.53 |  | 85.72 | 85.86 | 85.93 |
| *S. suis* 165 | 12 | 62.08 | 62.00 | 60.34 |  | 83.91 | 83.42 | 83.88 |
| *S. suis* 170 | 12 | 31.47 | 28.48 | 29.87 |  | 75.14 | 75.22 | 75.71 |
| *S. suis* 4972 1C | 12 | 28.17 | 29.41 | 28.49 |  | 75.23 | 75.48 | 75.28 |
| *S. suis* 4993 11B | 12 | 20.36 | 20.92 | 20.38 |  | 75.65 | 75.81 | 75.54 |
| *S. suis* 5171 2C | 12 | 40.13 | 41.14 | 40.46 |  | 83.63 | 82.84 | 83.71 |
| *S. suis* 5171 6B | 12 | 32.14 | 33.23 | 32.63 |  | 82.13 | 81.92 | 81.29 |
| *S. suis* 5278 16B | / | 30.99 | 33.24 | 30.06 |  | 76.30 | 76.33 | 76.23 |
| *S. suis* 5321 1B | 12 | 55.42 | 56.80 | 55.01 |  | 67.79 | 67.20 | 67.01 |
| *S. suis* EN2 | 12 | 36.64 | 36.19 | 37.20 |  | 57.73 | 58.90 | 58.93 |
| *S. suis* EN15 | 16 | 36.50 | 34.86 | 35.21 |  | 85.22 | 84.72 | 84.69 |
| *S. suis* EN38 | 12 | 23.79 | 25.35 | 23.20 |  | 67.52 | 68.07 | 67.49 |
| *S. suis* EN39 | 12 | 24.78 | 26.51 | 25.71 |  | 66.59 | 66.96 | 66.06 |
