# Supplementary material for: Development of a novel chimeric lysin to combine parental phage lysin and cefquinome for preventing sow endometritis after artificial insemination
Source: Vet Res. 2025 Feb 11;56:39. doi: 10.1186/s13567-025-01457-4 (PMC11816537; doi:10.1186/s13567-025-01457-4)
Supplement: Supplementary file 9 — Additional file 9. Turbidity decrease caused by the phage lysins ClyL and Lys0859 against Staphylococcus. [file 13567_2025_1457_MOESM9_ESM.doc]

**Additional file 9 The turbidity decrease of phage lysin ClyL and Lys0859 against *Staphylococcus*.**

| Strains | Decrease of turbidity/% | | | | | | |
| --- | --- | --- | --- | --- | --- | --- | --- |
|  | ClyL | | |  | Lys0859 | | |
| *Staphylococcus* 1 | 51.65 | 49.56 | 50.64 |  | 24.45 | 20.91 | 22.73 |
| *Staphylococcus* 2 | 57.45 | 57.43 | 57.88 |  | 20.56 | 19.83 | 21.30 |
| *Staphylococcus* 3 | -0.14 | 0.25 | 3.53 |  | 0.00 | 2.09 | 1.43 |
| *Staphylococcus* 4 | 50.82 | 50.21 | 49.12 |  | 27.93 | 27.10 | 23.17 |
| *Staphylococcus* 5 | 43.43 | 41.88 | 42.26 |  | 25.83 | 26.18 | 26.49 |
| *Staphylococcus* 6 | 44.99 | 46.86 | 45.91 |  | 22.61 | 21.83 | 22.22 |
| *Staphylococcus* 7 | 37.59 | 37.77 | 38.27 |  | 27.43 | 27.91 | 28.08 |
| *Staphylococcus* 8 | 45.92 | 46.33 | 45.91 |  | 37.36 | 38.60 | 36.75 |
| *Staphylococcus* 9 | 39.94 | 41.10 | 39.41 |  | 26.17 | 25.81 | 22.98 |
| *Staphylococcus* 10 | 58.99 | 58.82 | 59.25 |  | 28.95 | 30.96 | 31.46 |
| *Staphylococcus* 11 | 79.93 | 79.02 | 80.06 |  | 66.58 | 68.72 | 69.04 |
| *Staphylococcus* 12 | 34.76 | 34.99 | 34.88 |  | 13.89 | 13.61 | 13.75 |
| *Staphylococcus* 13 | 62.30 | 62.35 | 61.05 |  | 40.20 | 40.71 | 39.66 |
| *Staphylococcus* 14 | 31.50 | 32.05 | 31.72 |  | 19.84 | 19.70 | 17.76 |
| *Staphylococcus* 15 | 45.93 | 46.55 | 48.14 |  | 5.86 | 6.16 | 5.41 |
| *Staphylococcus* 16 | 29.07 | 26.51 | 27.79 |  | 11.51 | 12.74 | 12.13 |
| *Staphylococcus* 17 | 24.54 | 24.52 | 24.53 |  | 8.06 | 6.78 | 7.42 |
| *Staphylococcus* 18 | 24.02 | 23.44 | 23.73 |  | 7.25 | 6.26 | 6.76 |
| *Staphylococcus* 19 | 46.33 | 46.07 | 46.20 |  | 18.06 | 16.93 | 17.50 |
| *Staphylococcus* 20 | 37.65 | 36.34 | 37.42 |  | 21.28 | 20.12 | 21.15 |
| *Staphylococcus* 21 | 63.30 | 62.81 | 62.68 |  | 44.49 | 42.53 | 42.76 |
| *Staphylococcus* 22 | 47.68 | 49.12 | 48.52 |  | 24.60 | 26.23 | 24.52 |
| *Staphylococcus* 23 | 0.86 | 0.63 | 0.51 |  | 1.24 | 2.94 | 2.71 |
| *Staphylococcus* 25 | 19.88 | 20.52 | 20.20 |  | 5.69 | 5.71 | 5.70 |
| *Staphylococcus* 26 | 22.43 | 23.02 | 22.72 |  | 6.15 | 6.39 | 6.27 |
| *Staphylococcus* 27 | 23.18 | 24.82 | 24.68 |  | 7.47 | 10.65 | 10.40 |
| *Staphylococcus* 28 | 47.22 | 46.93 | 47.08 |  | 24.63 | 25.00 | 24.81 |
| *Staphylococcus* 29 | 47.98 | 47.20 | 47.59 |  | 18.93 | 18.57 | 18.75 |
| *Staphylococcus* 30 | 79.26 | 78.07 | 76.72 |  | 63.54 | 62.86 | 61.21 |
| *Staphylococcus* 32 | 1.64 | 3.55 | 3.19 |  | 2.67 | 4.35 | 2.25 |
| *Staphylococcus* 33 | 42.11 | 41.70 | 40.85 |  | 9.87 | 6.89 | 8.35 |
| *Staphylococcus* 34 | 57.75 | 56.23 | 56.99 |  | 32.03 | 30.24 | 31.13 |
| *Staphylococcus* 35 | 39.88 | 38.86 | 39.37 |  | 17.86 | 19.99 | 18.93 |
| *Staphylococcus* 36 | 70.87 | 71.67 | 71.21 |  | 32.62 | 33.63 | 34.29 |
| *Staphylococcus* 37 | 70.86 | 71.69 | 72.42 |  | 53.08 | 52.62 | 51.98 |
| *Staphylococcus* 38 | 41.69 | 42.28 | 41.99 |  | 18.71 | 19.25 | 18.98 |
| *Staphylococcus* 39 | 2.21 | -0.65 | -0.18 |  | 0.34 | 0.53 | 1.73 |
| *Staphylococcus* 40 | 2.50 | 3.95 | 4.42 |  | 1.73 | 1.18 | 0.00 |
| *Staphylococcus* 41 | 0.68 | 4.35 | 4.10 |  | -0.76 | 1.07 | 2.64 |
| *Staphylococcus* 42 | 40.45 | 40.00 | 40.25 |  | 18.26 | 16.56 | 16.80 |
| *Staphylococcus* 43 | 50.13 | 49.05 | 49.59 |  | 24.32 | 21.71 | 23.02 |
| *Staphylococcus* 44 | 39.78 | 39.90 | 39.84 |  | 19.92 | 19.18 | 19.55 |
| *Staphylococcus* 45 | 2.13 | 2.64 | 0.85 |  | -0.17 | 0.02 | 0.57 |
| *Staphylococcus* 46 | 3.72 | -2.99 | 4.57 |  | 0.54 | -2.96 | 3.36 |
| *Staphylococcus* 47 | 3.21 | 6.42 | 5.33 |  | -2.59 | 1.39 | 2.14 |
| *Staphylococcus* 48 | 6.18 | 6.15 | 13.15 |  | 4.99 | 1.25 | 5.95 |
| *Staphylococcus* 49 | -1.84 | 1.64 | 3.80 |  | -1.43 | 3.13 | -0.22 |
| *Staphylococcus* 50 | 67.59 | 67.67 | 66.62 |  | 58.12 | 57.46 | 56.53 |
| *Staphylococcus* 51 | 5.41 | 4.43 | 3.47 |  | -0.19 | 1.40 | 2.24 |
| *Staphylococcus* 52 | 79.48 | 79.71 | 80.96 |  | 77.77 | 77.55 | 77.22 |
| *Staphylococcus* 53 | 51.10 | 52.07 | 51.58 |  | 17.69 | 17.27 | 17.48 |
| *Staphylococcus* 54 | 54.72 | 55.07 | 55.02 |  | 22.24 | 23.48 | 24.19 |
| *Staphylococcus* 55 | 18.30 | 19.76 | 21.30 |  | 1.21 | -0.70 | 0.93 |
| *Staphylococcus* 57 | 2.64 | 5.97 | 5.22 |  | 0.23 | 3.09 | 2.27 |
| *Staphylococcus* 58 | 41.83 | 41.94 | 41.89 |  | 21.89 | 21.69 | 21.79 |
| *Staphylococcus* 59 | 73.35 | 72.42 | 73.34 |  | 60.87 | 59.98 | 60.61 |
| *Staphylococcus* 60 | 0.11 | -1.27 | 1.26 |  | 1.39 | 1.87 | 1.09 |
| *Staphylococcus* 61 | 26.70 | 27.68 | 27.19 |  | 12.74 | 11.58 | 12.16 |
| *Staphylococcus* 62 | 38.15 | 36.88 | 38.56 |  | 12.44 | 10.83 | 8.85 |
| *Staphylococcus* 63 | 46.22 | 46.88 | 46.91 |  | 36.37 | 39.42 | 39.07 |
| *Staphylococcus* 64 | 42.80 | 42.46 | 42.63 |  | 15.14 | 14.25 | 14.69 |
| *Staphylococcus* 65 | 63.42 | 62.76 | 63.09 |  | 32.12 | 33.88 | 33.00 |
| *Staphylococcus* 67 | 61.44 | 62.71 | 61.17 |  | 54.83 | 51.95 | 53.59 |
| *Staphylococcus* 68 | 69.55 | 69.92 | 69.73 |  | 43.39 | 44.32 | 43.86 |
| *Staphylococcus* 70 | 10.47 | 10.24 | 12.26 |  | 0.04 | 0.02 | 1.61 |
| *Staphylococcus* 71 | 51.81 | 50.64 | 51.23 |  | 18.88 | 18.28 | 18.58 |
| *Staphylococcus* 72 | 70.40 | 70.98 | 70.69 |  | 7.80 | 8.19 | 8.00 |
| *Staphylococcus* 74 | 52.34 | 51.35 | 51.98 |  | 13.42 | 13.49 | 13.13 |
| *Staphylococcus* 76 | 28.69 | 30.14 | 29.42 |  | 12.96 | 14.59 | 13.78 |
| *Staphylococcus* 77 | 27.45 | 28.65 | 28.05 |  | 13.83 | 12.73 | 13.28 |
| *Staphylococcus* 78 | 33.40 | 33.31 | 33.35 |  | 16.84 | 17.98 | 17.42 |
| *Staphylococcus* 79 | 33.01 | 34.55 | 33.78 |  | 16.39 | 16.38 | 16.38 |
| *Staphylococcus* 80 | 28.69 | 27.04 | 27.86 |  | 14.61 | 12.42 | 13.51 |
| *Staphylococcus* 82 | 9.25 | 8.42 | 10.81 |  | 0.00 | -0.31 | 1.35 |
| *Staphylococcus* 84 | 77.94 | 76.56 | 77.82 |  | 68.62 | 67.62 | 68.40 |
| *Staphylococcus* 85 | 43.11 | 42.94 | 41.80 |  | 30.91 | 30.65 | 30.89 |
| *Staphylococcus* 86 | 63.19 | 63.35 | 64.48 |  | 29.10 | 28.01 | 30.17 |
| *Staphylococcus* 87 | 54.12 | 55.17 | 54.64 |  | 24.93 | 26.21 | 25.56 |
| *Staphylococcus* 88 | 63.25 | 63.55 | 61.92 |  | 58.09 | 58.81 | 59.79 |
| *Staphylococcus* 89 | 38.33 | 38.34 | 37.69 |  | 29.29 | 28.32 | 28.11 |
| *Staphylococcus* 90 | 20.58 | 19.85 | 20.34 |  | 6.33 | 6.19 | 6.69 |
| *Staphylococcus* 91 | 34.45 | 34.63 | 34.54 |  | 18.25 | 19.28 | 18.76 |
| *Staphylococcus* 92 | 43.98 | 42.29 | 43.15 |  | 18.36 | 18.86 | 18.61 |
| *Staphylococcus* 93 | 50.14 | 51.17 | 50.65 |  | 21.64 | 19.99 | 20.82 |
| *Staphylococcus* 94 | 57.36 | 57.54 | 58.26 |  | 53.67 | 53.71 | 55.22 |
| *Staphylococcus* 95 | 43.40 | 43.49 | 43.44 |  | 22.25 | 20.51 | 21.38 |
| *Staphylococcus* 96 | 56.12 | 55.66 | 54.42 |  | 47.19 | 45.16 | 46.40 |
| *Staphylococcus* 97 | 15.03 | 14.79 | 14.91 |  | 3.09 | 4.16 | 3.63 |
| *Staphylococcus* 98 | 56.62 | 54.54 | 58.32 |  | 17.80 | 9.51 | 18.34 |
| *Staphylococcus* 99 | 53.84 | 54.37 | 54.11 |  | 22.69 | 27.55 | 25.14 |
| *Staphylococcus* 100 | 60.35 | 59.89 | 59.64 |  | 26.23 | 27.34 | 29.18 |
| *Staphylococcus* 101 | 68.96 | 68.84 | 66.12 |  | 33.03 | 33.92 | 28.90 |
| *Staphylococcus* 102 | 69.62 | 69.25 | 69.44 |  | 44.94 | 42.71 | 43.83 |
| *Staphylococcus* 103 | 43.96 | 46.62 | 46.83 |  | 9.31 | 10.90 | 12.32 |
| *Staphylococcus* 104 | 57.03 | 58.67 | 57.85 |  | 29.29 | 29.05 | 29.17 |
| *Staphylococcus* 105 | 50.47 | 50.64 | 50.29 |  | 16.59 | 17.08 | 16.51 |
| *Staphylococcus* 106 | 61.99 | 61.43 | 62.36 |  | 36.48 | 39.03 | 40.06 |
| *Staphylococcus* 109 | 60.79 | 59.52 | 60.16 |  | 36.48 | 36.49 | 36.48 |
| *Staphylococcus* 110 | 52.06 | 51.94 | 52.00 |  | 19.77 | 23.53 | 21.67 |
| *Staphylococcus* 111 | 23.00 | 25.21 | 24.12 |  | 7.96 | 10.75 | 9.37 |
| *Staphylococcus* 112 | 54.97 | 55.94 | 55.26 |  | 26.54 | 24.79 | 25.78 |
| *Staphylococcus* 113 | 68.16 | 68.59 | 66.56 |  | 35.43 | 37.25 | 37.50 |
| *Staphylococcus* 114 | 67.18 | 67.85 | 68.81 |  | 39.41 | 39.54 | 38.66 |
| *Staphylococcus* 115 | 58.50 | 59.41 | 59.16 |  | 40.58 | 43.13 | 42.89 |
| *Staphylococcus* 117 | 37.86 | 42.42 | 40.15 |  | 20.98 | 20.43 | 20.71 |
| *Staphylococcus* 118 | 56.32 | 57.92 | 57.12 |  | 29.67 | 30.28 | 29.97 |
| *Staphylococcus* 119 | 68.39 | 69.10 | 68.75 |  | 43.08 | 44.10 | 43.60 |
| *Staphylococcus* 120 | 50.12 | 51.23 | 50.67 |  | 21.91 | 23.47 | 22.69 |
| *Staphylococcus* 121 | 49.00 | 48.84 | 48.92 |  | 17.48 | 18.16 | 17.82 |
| *Staphylococcus* 122 | 56.80 | 56.31 | 56.56 |  | 6.40 | 6.23 | 6.32 |
| *Staphylococcus* 124 | 3.77 | 3.55 | 3.66 |  | 2.91 | 2.33 | 2.62 |
| *Staphylococcus* 125 | 11.91 | 13.06 | 12.49 |  | 6.93 | 7.66 | 7.30 |
| *Staphylococcus* 126 | 66.86 | 65.43 | 66.16 |  | 44.01 | 41.72 | 42.89 |
| *Staphylococcus* 127 | 59.20 | 61.98 | 60.60 |  | 25.24 | 18.79 | 22.01 |
| *Staphylococcus* 128 | 47.49 | 49.20 | 48.35 |  | 20.40 | 20.77 | 20.59 |
| *Staphylococcus* 129 | 55.99 | 56.34 | 56.17 |  | 25.21 | 27.55 | 26.38 |
| *Staphylococcus* 130 | 78.76 | 78.77 | 78.77 |  | 19.23 | 18.17 | 18.70 |
| *Staphylococcus* 131 | 61.24 | 61.44 | 61.34 |  | 19.37 | 19.07 | 19.22 |
| *Staphylococcus* 132 | 26.93 | 26.29 | 26.61 |  | 9.39 | 8.38 | 8.88 |
| *Staphylococcus* 134 | 40.56 | 43.60 | 42.08 |  | 27.31 | 27.12 | 27.22 |
| *Staphylococcus* 135 | 26.15 | 25.31 | 25.73 |  | 13.99 | 11.75 | 12.87 |
| *Staphylococcus* 138 | 52.12 | 53.88 | 53.00 |  | 29.53 | 29.28 | 29.40 |
| *Staphylococcus* 141 | 38.80 | 39.84 | 39.32 |  | 17.38 | 15.60 | 16.49 |
| *Staphylococcus* 142 | 44.24 | 47.32 | 45.80 |  | 24.11 | 22.66 | 23.37 |
| *Staphylococcus* 143 | 4.47 | 7.83 | 6.18 |  | 4.33 | 8.01 | 6.20 |
| *Staphylococcus* 144 | 35.62 | 35.32 | 35.47 |  | 17.53 | 16.32 | 16.93 |
| *Staphylococcus* 145 | 54.75 | 54.20 | 54.48 |  | 18.36 | 18.64 | 18.50 |
| *Staphylococcus* 146 | 39.48 | 40.77 | 40.14 |  | 18.05 | 18.70 | 18.38 |
| *Staphylococcus* 147 | 52.01 | 52.79 | 52.40 |  | 24.66 | 21.70 | 23.17 |
| *Staphylococcus* 148 | 68.22 | 67.81 | 68.02 |  | 40.76 | 40.53 | 40.65 |
| *Staphylococcus* 150 | 67.53 | 65.49 | 66.52 |  | 21.24 | 11.64 | 16.48 |
| *Staphylococcus* 151 | 52.11 | 50.70 | 51.41 |  | 18.20 | 9.69 | 13.98 |
| *Staphylococcus* 152 | 70.74 | 71.46 | 71.11 |  | 17.27 | 20.97 | 19.15 |
| *Staphylococcus* 153 | 47.33 | 45.62 | 46.49 |  | 22.55 | 21.90 | 22.23 |
| *Staphylococcus* 154 | 22.92 | 19.94 | 21.44 |  | 10.17 | 9.29 | 9.73 |
| *Staphylococcus* 155 | 2.55 | 1.48 | 2.02 |  | 4.11 | 3.62 | 3.87 |
| *Staphylococcus* 156 | 71.24 | 71.97 | 71.60 |  | 24.62 | 26.76 | 25.70 |
| *Staphylococcus* 157 | 60.07 | 59.88 | 59.97 |  | 32.80 | 33.25 | 33.03 |
| *Staphylococcus* 158 | 29.89 | 30.50 | 30.19 |  | 7.06 | 5.60 | 6.33 |
| *Staphylococcus* 159 | 35.55 | 36.88 | 36.01 |  | 25.36 | 25.25 | 24.82 |
| *Staphylococcus* 160 | 44.46 | 43.46 | 42.19 |  | 37.27 | 35.39 | 36.88 |
