# Supplementary material for: Development of a novel chimeric lysin to combine parental phage lysin and cefquinome for preventing sow endometritis after artificial insemination
Source: Vet Res. 2025 Feb 11;56:39. doi: 10.1186/s13567-025-01457-4 (PMC11816537; doi:10.1186/s13567-025-01457-4)
Supplement: Supplementary file 10 — Additional file 10. FIC indices for the combinations of ClyL and Lys0859. [file 13567_2025_1457_MOESM10_ESM.doc]

**Additional file 10 The FIC index for the combinations of ClyL and Lys0859**.

| Strains | MIC (ug/mL) | | | Lowest FICI |
| --- | --- | --- | --- | --- |
|  | ClyL | Lys0859 | ClyL+Lys0859 |  |
| *S. agalactiae* ATCC13813 | 128 | 16 | 2/4 | 0.266 |
| *S. aureus* ATCC29213 | 16 | 64 | 4/16 | 0.5 |
| *Staphylococcus* st65 | 8 | 16 | 0.125/4 | 0.266 |
| *S. agalactiae* ATCC13813+*S. aureus* ATCC29213 | 4 | 64 | 1/2 | 0.281 |
