# Supplementary figures and images for: Development of a novel chimeric lysin to combine parental phage lysin and cefquinome for preventing sow endometritis after artificial insemination
Source: Vet Res. 2025 Feb 11;56:39. doi: 10.1186/s13567-025-01457-4 (PMC11816537; doi:10.1186/s13567-025-01457-4)

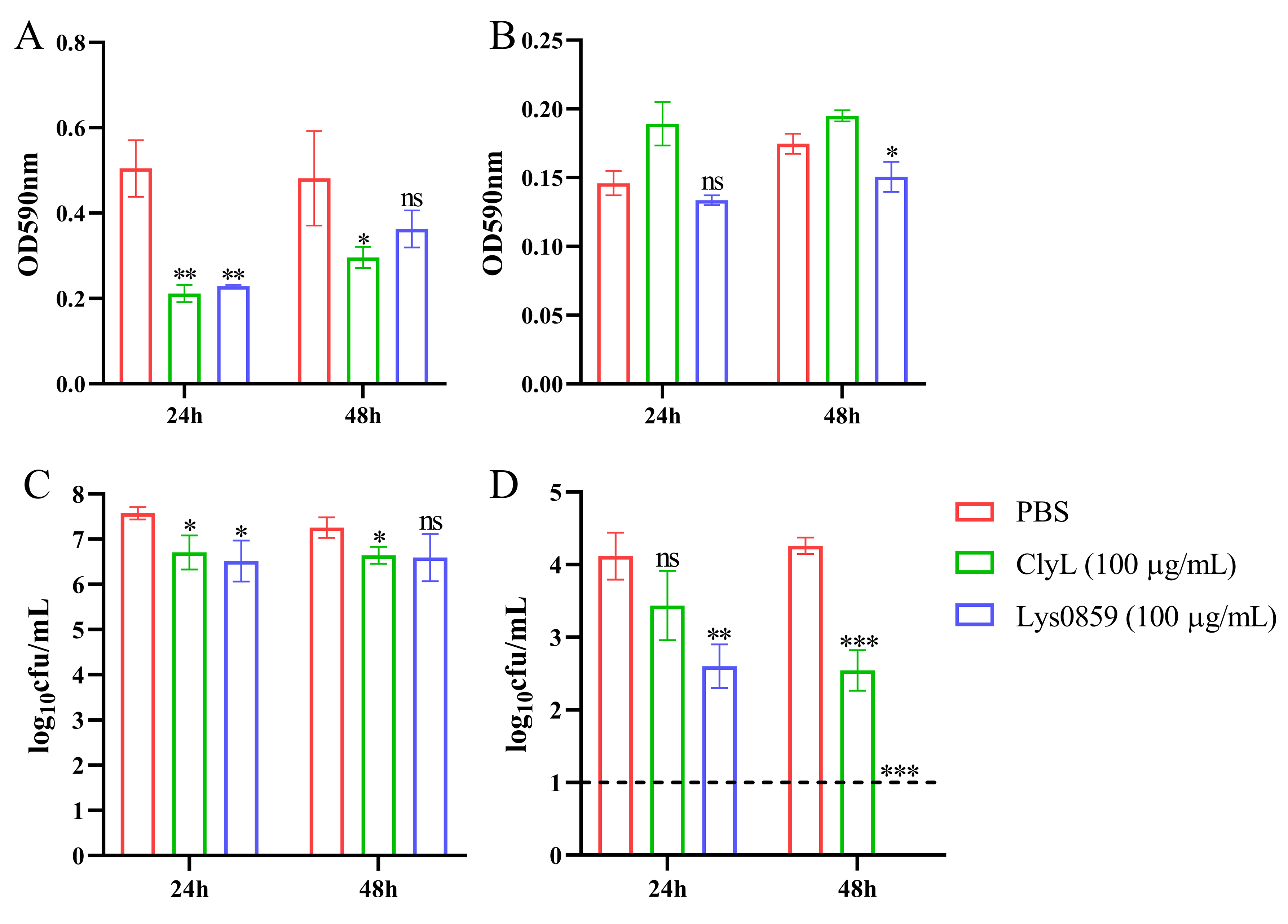

Supplement: Supplementary file 11 — Additional file 11. Chimeric lysin ClyL and parental phage lysin Lys0859-treated 24-h- and 48-h-old biofilms formed from single S. aureus ATCC29213 and S. agalactiae ATCC13813 strains. The biomass of single S. aureus ATCC29213 (A) and S. agalactiae ATCC13813 (B) biofilms was treated with ClyL and Lys0859 at 37 °C for 2 h. The viable cell counts of single S. aureus ATCC29213 (C) and S. agalactiae ATCC13813 (D) biofilms were determined after treatment with ClyL and Lys0859 at 37 °C for 2 h. Significant differences between the PBS groups and the ClyL and Lys0859 groups were determined by Student’s t test (ns p > 0.05, * p < 0.05 ** p < 0.01, and *** p < 0.001). The experiments were performed three times. The data are shown as the mean ± SD. [file 13567_2025_1457_MOESM11_ESM.doc]
