# Supplementary material for: Development of a novel chimeric lysin to combine parental phage lysin and cefquinome for preventing sow endometritis after artificial insemination
Source: Vet Res. 2025 Feb 11;56:39. doi: 10.1186/s13567-025-01457-4 (PMC11816537; doi:10.1186/s13567-025-01457-4)
Supplement: Supplementary file 12 — Additional file 12. Effects of ClyL, Lys0859 and cefquinome on various kinematic parameters (motility) of sperm. [file 13567_2025_1457_MOESM12_ESM.doc]

**Additional file 12 Effect of ClyL, Lys0859 and cefquinome on the various kinematic parameters (motility) of sperm**.

| **Time** **(day)** | **Groups** | **VCL** | **VSL** | **VAP** | **LIN** | **WOB** | **ALH** | **BCF** |
| --- | --- | --- | --- | --- | --- | --- | --- | --- |
|  |  | **(μm/s)** | **(μm/s)** | **(μm/s)** | **(%)** | **(%)** | **(μm)** | **(Hz)** |
| Day 4 | Control | 182.1 ± 3.3 | 37.1 ± 2.9 | 84.77 ± 1.24 | 22.7 ± 2.92 | 46.43 ± 3.4 | 9.9 ± 0.4 | 35.4 ± 0.8 |
|  | ClyL (25 mg/mL) + Lys0859 (12.5 mg/mL) | 186.6 ± 1.5 | 37.2 ± 0.7 | 85.9 ± 0.8 | 22.97 ± 2.39 | 46.1 ± 0.6 | 9.7 ± 0.5 | 34.2 ± 0.1 |
|  | Cefquinome (80 mg/mL) | 191.87 ± 3.71 | 37.4 ± 0.4 | 88.6 ± 0.6 | 22.4 ± 2.69 | 46.47 ± 2.45 | 10.1 ± 0.6 | 34.5 ± 0.4 |
|  | ClyL (25 mg/mL) +Lys0859 (12.5 mg/mL) +Cefquinome (80 mg/ml) | 187.9 ± 5.1 | 35.8 ± 1.3 | 83.5 ± 1.52 | 23.73 ± 2.65 | 47.43 ± 2.15 | 9.57 ± 0.5 | 34.7 ± 0.5 |
| Day 8 | Control | 130.17 ± 3.55 | 36.6 ± 0.53 | 62.17 ± 1.5 | 30.97 ± 1 | 47.67 ± 1.65 | 8.83 ± 0.31 | 37.2 ± 1.15 |
|  | ClyL (25 mg/mL) + Lys0859 (12.5 mg/mL) | 129.33 ± 4.12 | 35.07 ± 2.96 | 61.87 ± 2.85 | 31 ± 1.67 | 47.97 ± 1 | 8.43 ± 0.68 | 37.7 ± 2.5 |
|  | Cefquinome (80 mg/mL) | 125.9 ± 3.7 | 34.17 ± 4 | 60.53 ± 1.5 | 32.37 ± 2.51 | 47.8 ± 2.55 | 8 ± 0.46 | 37.6 ± 3.5 |
|  | ClyL (25 mg/mL) +Lys0859 (12.5 mg/mL) +Cefquinome (80 mg/ml) | 131.07 ± 2.55 | 36.53 ± 1.95 | 62.5 ± 2.46 | 30.17 ± 2.54 | 47.57 ± 2.7 | 8.77 ± 0.75 | 37.13 ± 1.98 |
| Day 12 | Control | 111.43 ± 3.4 | 24.9 ± 0.95 | 49.03 ± 2.22 | 24.63 ± 1.76 | 41.4 ± 1.51 | 7.63 ± 0.45 | 40.97 ± 1.5 |
|  | ClyL (25 mg/mL) + Lys0859 (12.5 mg/mL) | 109.23 ± 3.25 | 25.4 ± 2.17 | 50.07 ± 2.46 | 24.23 ± 2.2 | 42.53 ± 1.5 | 7.47 ± 0.64 | 41.27 ± 2.05 |
|  | Cefquinome (80 mg/mL) | 115.7 ± 2.52 | 26.9 ± 2.4 | 51.57 ± 1.6 | 26.23 ± 3.03 | 41.5 ± 0.5 | 8.3 ± 0.3 | 37.47 ± 1.42 |
|  | ClyL (25 mg/mL) +Lys0859 (12.5 mg/mL) +Cefquinome (80 mg/mL) | 114.23 ± 2.53 | 26.3 ± 1.5 | 50.77 ± 2.25 | 25.03 ± 2.76 | 42.13 ± 1.15 | 8.33 ± 0.55 | 39.23 ± 2.04 |
